# Valorization of Agro-Industrial By-Products in the Development of Bio-Polymer Films, Biodegradable Plasticulture, and Intelligent Food-Packaging Materials

**DOI:** 10.3390/polym18192343

**Published:** 2026-09-25

**Authors:** Ganesh Saratale, Rijuta Ganesh Saratale, Ramesh Kumar, Han Seung Shin, Byong-Hun Jeon

**Affiliations:** 1 Department of Food Science and Biotechnology, Dongguk University-Seoul, Ilsandong-gu, Goyang-si 10326, Gyeonggi-do, Republic of Koreaspartan@dongguk.edu (H.S.S.); 2 Department of Earth Resources & Environmental Engineering, Hanyang University, Seoul 04763, Republic of Korea; cherryg2310@gmail.com (R.G.S.); rameshibt@gmail.com (R.K.)

**Keywords:** agro-industrial by-products, biopolymers, biodegradable plasticulture, yerba mate, topinambur, red cabbage anthocyanins, intelligent packaging, circular economy

## Abstract

The increasing environmental impact of petroleum-based plastics has intensified the need for renewable, biodegradable, active, and intelligent materials for food packaging and agricultural plasticulture. This review critically analyzes the potential to valorize agro-industrial by-products and underutilized biomass to produce functional biodegradable materials by extracting biopolymers, reinforcing fillers, bioactive compounds, and natural pigments. Particular emphasis is placed on starch-rich and fructan-rich crops, such as cassava, potato, sweet potato, and Jerusalem artichoke, or topinambur (*Helianthus tuberosus*), which are potential raw materials for the manufacture of biodegradable films, coatings, and agricultural mulches. The multifunctional use of yerba-mate residues (*Ilex paraguariensis*) as reinforcement fillers, antioxidant additives, and pH-responsive components in biopolymer composites is also discussed. Rosehip (*Rosa rubiginosa*), *Aloe vera* (*Aloe barbadensis*), and Jerusalem artichoke are also described as sources of polysaccharides, dietary fibers, phenolic compounds, and natural colorants that can enhance the mechanical, barrier, antioxidant, and functional properties of the biodegradable materials. Recent developments are discussed, including freshness-monitoring packaging systems containing pH-sensitive anthocyanins extracted from red cabbage and the promising application of pigments from Jerusalem artichoke flowers. In addition, the review identifies the main challenges for industrial application, such as mechanical and barrier performance, moisture sensitivity, the instability of natural pigments and bioactive compounds, and the control of biodegradability. It also highlights challenges related to food safety control, regulatory compliance, raw-material availability, and scalability. In general, agro-industrial biomass valorization, combined with biodegradable polymer processing, active capabilities, and intelligent sensing technologies, offers a promising path toward partially replacing conventional plastics and improving resource efficiency in sustainable food-packaging and plasticulture systems integrated into a circular bioeconomy. Unlike many previous reviews, which treat biodegradable food packaging, intelligent packaging, and agricultural mulch film as separate research fields, this review integrates these applications into a unified circular-bioeconomy approach based on agro-industrial biomass valorization. It presents a unique approach that compares feedstock composition, processing needs, material and functional properties, application suitability, and industrial viability with regard to food-packaging systems and plasticulture systems. In this review, the authors connect biodegradable structural materials and their active and intelligent functions with end-of-life requirements for specific applications. This combined perspective for developing multifunctional agro-waste-derived materials offers better chances for industrial translation.

## 1. Introduction

Over the last few decades, the demand for plastic materials has greatly increased worldwide due to rapid industrialization, urbanization, and improvements in living standards. Petroleum-derived plastics have become indispensable in many industrial applications due to their strong mechanical properties, flexibility, durability, low cost of manufacture, and outstanding thermal and barrier properties. They are widely used in packaging, agriculture, healthcare, construction, transportation, and consumer goods [1,2]. Consequently, cumulative global plastic production has exceeded 9 billion tonnes, and today’s society is increasingly dependent on fossil fuel-derived polymers. Although conventional plastics provide substantial economic value, their production and consumption are nonetheless responsible for one of the most serious environmental issues of the twenty-first century. Most petroleum-based plastics are highly resistant to degradation by microorganisms; enormous quantities of plastic waste have been found in terrestrial and aquatic environments, resulting in increased carbon footprints, greenhouse gas emissions, biodiversity loss, soil and marine pollution, as well as the formation of persistent microplastics and nanoplastics that pose risks to human health and have negative effects on ecosystem integrity [3]. If no measures are taken, global plastic waste could nearly double by 2030, further increasing environmental pollution and creating pressure on strained waste-management systems [4,5]. The food-packaging and agriculture sectors are two major sectors of plastic use, where plastic materials successfully protect food from physical injury, moisture, oxygen, light, and microorganisms, extend shelf life, reduce food waste, and enable food transportation and storage [6]. Likewise, plastics are also used in the field of agriculture in myriad ways, such as mulch films, greenhouse covers, irrigation systems, mulch wrap for silage, seedling trays, and controlled-release formulations, which significantly improve productivity and resource-use efficiency [7,8]. Given all these essential functions, the complete elimination of plastics is impractical. The main challenge is to develop environmentally friendly materials and replace traditional fossil-based materials with them, while achieving comparable performance with a much smaller environmental footprint.

Petroleum-based polymers in packaging protect foods from oxygen, water, microbial contamination, and mechanical damage. In agriculture, plasticulture technologies like mulch films, greenhouse covers, irrigation tubing, seedling trays, and protective cover tunnels have improved crop productivity, water-use efficiency, weed suppression, and soil-temperature management. However, the advantages provided by these technologies are accompanied by significant environmental trade-offs [6]. The global plastic system is linked to greenhouse-gas emissions, fossil resource dependence, and long-term accumulation of waste as products move into terrestrial and aquatic ecosystems. The plastic life cycle can significantly contribute to global greenhouse gas (GHG) emissions, and agricultural and food-packaging chains use large amounts of plastic products annually [9,10]. Conventional plastics are of particular concern because they remain a major fraction of plastic waste, which ends up in landfills or is burnt, lost, or fragmented into microplastics [11]. Agricultural plastic films are particularly challenging to retrieve after use because they are thin, soil-contaminated, and susceptible to tearing during harvest. Biopolymers and bio-based composites provide viable pathways to reduce reliance on fossil fuel-based plastics. Films, coatings, foams, fibers, hydrogels, and composites can be produced from polysaccharides, proteins, lipids, lignocellulosic fibers, and biodegradable polyesters [12]. Sustainability depends not only on bio-based origins but also on processing needs, functionality, environmental fate, end-of-life options, and life-cycle impacts. Biodegradability should be carefully examined, as degradation time and rates depend on polymer chemistry, crystallinity, morphology, additives, microbial community, temperature, moisture, oxygen, and soil/compost conditions [13,14]. With this in mind, agro-industrial waste can be considered as a potential feedstock for sustainable polymeric materials. Significant amounts of peels, pomace, spent leaves, tuber residues, seed cakes, extraction residues, fibers, flowers, and stems are produced by food and agricultural industries. These streams are rich in starch, cellulose, hemicellulose, pectin, inulin, proteins, oils, phenolics, anthocyanins, tannins, and other valuable compounds. Valorizing their residues can lead to the production of functional packaging materials, as well as materials for plasticulture, in line with a circular economy framework [14,15,16].

This challenge has stimulated global research trends to develop renewable, biodegradable, and circular polymers from renewable resources. Significant efforts have been devoted to the valorization of biomass from agriculture, food-processing by-products, microbial polymers, algae, and other bio-renewable materials, as sustainable alternatives to petrochemical plastics. Bio-based materials offer advantages beyond reducing dependence on finite fossil resources, as they also enable circular bio-economy strategies by turning renewable biomass and agro-industrial by-products into functional added-value products, thus reducing waste production, greenhouse gas emissions, and the use of fossil resources through efficient use of materials. In this context, several promising classes of sustainable materials have been identified, namely bioplastics [1,2,7]. Bioplastics, according to their origin and composition, can be bio-based or biodegradable or can possess both characteristics. The physicochemical, mechanical, and processing properties of many commercially available and emerging bioplastics are similar to those of conventionally produced petroleum-based plastics, making them suitable for use in a variety of applications, such as food packaging, agriculture, biomedical engineering, and consumer products [17]. Meanwhile, with the development of biomass valorization, green processing technologies, polymer blending, nanotechnology, and active and intelligent packaging, the performance, cost-effectiveness, and market acceptance of bioplastics are projected to improve further and facilitate the transition towards a more sustainable and circular material economy [4,5,6].

More recent reviews in 2026 reveal the transition from single petroleum plastic replacement to the design of multifunctional biopolymer systems by application. Reviews on the valorization of agro-industrial by-products tend to highlight the recovery of polymers, reinforcement materials, and bioactive products within an integrated approach, while also pointing out challenges such as feedstock variability, moisture sensitivity, and industrial validation [18,19]. More recent starch-focused reviews also indicate that blending, crosslinking, nanofillers, and plant extracts can be used in films to significantly enhance their performance, while issues related to hydrophilicity, formulation compatibility, scalability, and end-of-life behavior remain unresolved [20,21,22,23]. Recent reviews focus more on anthocyanin pH-response than on the development of intelligent packaging with multi-scale regulation mechanisms of both analyte transport and its response mechanisms, comprising pigment stability, matrix compatibility, response kinetics, and commercialization [24,25,26,27,28]. Similarly, recent reviews of plasticulture technology challenge the notion that biodegradable mulch films always have consistent environmental advantages, citing variability in field degradation and the creation of biodegradable microplastic residues [29]. These recent developments point to the need to optimize material performance, sensing reliability, process scalability, safety, and end-of-life behavior for specific applications.

The review examines the valorization of the by-products of industrial and agro-industrial processes as sustainable sources of bioactives and biopolymers. Special emphasis is given to roots and tubers for use in the formulation of biodegradable agronomic films, the use of yerba-mate by-products for antioxidant and pH-responsive composites, and other non-conventional biomass sources, such as rosehip, *Aloe vera*, and topinambur, used as feedstocks for functional materials. The natural pigments are used in active and intelligent packaging that responds to pH changes. In order to facilitate a description independent of materials, selected agro-industrial feedstocks are assessed under the background of a common analytical framework with six interrelated dimensions: (i) feedstock characteristics (thickness and composition of film-forming polymers, reinforcing fibers, bioactive compounds, and natural pigments); (ii) processing and fabrication requirements (extraction, blending, reinforcement, film-forming routes); (iii) material performance (mechanical, barrier, thermal, optical, moisture-related behavior); (iv) functional performance (anti-oxidant, antimicrobial, UV-protective and pH-responsive); (v) application suitability (food packaging, intelligent packaging, biodegradable plasticulture); and (vi) industrial feasibility (feedstock variability, process scalability, safety of industrial use, biodegradation, regulatory requirements, cost). This framework is used throughout the remainder of the document to identify the potential of biomass resources as well as their most important constraints, compromises and best applications in a circular bioeconomy.

## 2. Agro-Industrial By-Products as Circular Feedstocks for Polymeric Materials

The conversion of agro-industrial by-products to biodegradable polymeric materials is one of the most promising strategies for converting renewable feedstocks into materials that support the transition towards a circular bioeconomy and sustainable materials engineering. Millions of tons of agricultural and food-industry residues, such as fruit and vegetable peels, pomace, spent coffee grounds, spent brewery grains, cereal bran, husks, bagasse, stems, leaves, tuber residues, seed cakes, and extraction residues, are generated globally every year [30]. Instead of being discarded or used as low-value animal feed, these waste resources are now being converted into higher-value products like sustainable packaging materials and biocomposites, reducing reliance on fossil-based plastics and mitigating the environmental impacts linked to waste generation and disposal [14,31]. Newly published research highlights the importance of agro-industrial residues as multifunctional circular feedstocks that can be used simultaneously for structural reinforcement, bioactive functionality, and resource efficiency; as a result, they can play an important role in the development of modern bio-based materials [14,15,16,32,33]. Agro-industrial by-products are particularly valuable because of their high chemical diversity. Biomass residues contain multiple structural polymers and phytochemicals, with complementary functionalities. They are mostly composed of starch, cellulose, hemicelluloses, lignin, pectin, proteins, lipids, waxes, inulin, alginate, gums, phenolic acids, flavonoids, tannins, carotenoids, and anthocyanins [14,33,34]. Each component contributes to unique yet complementary functions during material fabrication. Biopolymers such as starch, pectin, proteins, inulin, and alginate have excellent film-forming ability and are mainly used as the polymer matrix or hydrocolloid network. Both cellulose and hemicellulose fractions are suggested to be reinforcing phases in polymer matrices, with cellulose contributing to tensile strength, stiffness, and dimensional stability and hemicellulose to improved mechanical properties, while lipids and waxes are hydrophobic components that reduce water-vapor permeability [35,36]. Numerous bioactive compounds, such as phenols, flavonoids, carotenoids, and anthocyanins, can impart antioxidant, antimicrobial, UV-protective, oxygen-scavenging, and pH-responsive properties, providing capabilities for active and intelligent packaging systems [14,32,33]. Thus, an agro-industrial residue can impart structural and functional properties to biodegradable polymeric materials.

Depending on the function involved in material design, the existing methods of biomass valorization can be broken down into three major strategies. The first strategy involves matrix formation, in which the continuous phase is composed of extracted biopolymers like starch (cassava, potato, maize, and sweet potato), pectin (fruit pomace), or polysaccharides (*Aloe vera*). Reinforcement engineering involves combining agricultural residues that have been broken down and ground to nanoscale dimensions (agro-nanomaterials) of lignocellulosic fibers, cellulose nanofibers (CNFs), and cellulose nanocrystals (CNCs) with biodegradable polymers, for increased mechanical strength, thermal stability, stiffness, opacity, and UV barrier properties, while reducing costs. The third strategy is bioactive functionalization, where bioactive extracts such as phenolic acids, essential oils, natural pigments, and antimicrobial metabolites isolated from agro-industrial residues are added to polymers to form packaging materials with antioxidant, antimicrobial, oxygen-scavenging, freshness-indicating, and pH-responsive properties [14,15,36]. Such complementary strategies highlight the ability of agro-industrial residues to act as functional bioactive reservoirs, polymer matrices, and reinforcing fillers. Methods for enhancing the fiber–matrix interfaces in biocomposites include treating the fibers to improve adhesion, using compatibilizers or anchors (coupling agents) to increase adhesion, and modifying the matrix to improve compatibility with the fibers. These strategies facilitate stress transfer, minimize debonding, and enhance mechanical properties [37]. These often include alkali treatment to remove surface impurities and increase surface texture for mechanical interlock. Additionally, silane coupling agents can generate chemical bridges between the fiber and the matrix, while plasma or corona treatment increases surface energy and introduces reactive groups. The use of compatibilizers like maleic anhydride-grafted polymers is also common to enhance bonding between hydrophilic fibers and hydrophobic matrices, as reported in [37,38,39].

Many recent innovations have resulted in new ways to utilize agro-industrial by-products, not just as basic, cost-effective fillers. Nanotechnology has facilitated the addition of cellulose nanofibers, cellulose nanocrystals, lignin nanoparticles, starch nanocrystals, and biochar (active carbon produced from organic wastes) to improve the properties of biodegradable composites, including tensile strength, oxygen-barrier performance, thermal stability, and moisture resistance [10]. On the other hand, there have been considerable advances in the use of green extraction techniques, such as ultrasound-assisted extraction (UAE), microwave-assisted extraction (MAE), enzyme-assisted extraction (EAE), supercritical fluid extraction (SFE), and deep eutectic solvent (DES)-based extraction, which have enhanced polysaccharide and bioactive compound yields, minimized solvent usage, and maintained the bioactivity of labile phytochemicals [14,16,31]. Further advances are related to the development from a passive, biodegradable film to an active, intelligent, and multifunctional packaging system. Grape pomace, citrus peel, fruit and vegetable residues, tea waste, spent coffee grounds, and herb-processing by-products contain a multitude of bioactive compounds and have become increasingly popular for inclusion in polymer films designed to reduce microbial proliferation and limit lipid oxidation, thereby prolonging food shelf life. In addition, hybrid composites (comprising agro-industrial residues and biodegradable polymers like PLA, PBAT, PBS, PHAs or chitosan, and nanoclays or nanocellulose) have shown synergistic and enhanced properties in terms of mechanical properties, gas barrier strength, thermal stability, antimicrobial activity and overall functionality, opening up the commercial potential of such hybrids [32,40]. Research has evolved from the immediate use of agricultural residues as a cheap filler to the rational design of multifunctional circular feedstocks where the same agro-industrial by-product is used as a polymer matrix, a reinforcing phase and a source of bioactive compounds. The continued development of more sustainable biomass valorization approaches, green extraction technologies, nanotechnology, materials engineering and digital optimization will lead to the development of next-generation biodegradable polymers to be used in food packaging, coatings, agricultural mulch films, biomedical materials and other sustainable applications, thus leading the transition towards the era of a circular and low-carbon bioeconomy [14,16,31,40]. Table 1 shows some examples of agro-industrial by-products that can be used as raw materials for biopolymers, reinforcing fillers, bioactive compounds, and ingredients for smart packaging. Likewise, anthocyanin-rich extracts from berry pomace, purple sweet potato, red cabbage, and other agricultural residues are used as natural pH-responsive freshness indicators owing to their reversible color changes under different spoilage conditions [2,6,7].

### 2.1. Starch Structure and Film Formation

Starch is a macromolecular semi-crystalline polysaccharide found to be made up of mostly linear amylose with α-(1 → 4) glycosidic linkages and highly branched amylopectin with α-(1 → 4) and α-(1 → 6) glycosidic linkages. The ratio of amylose to amylopectin, granule size, crystallinity, gelatinization temperature, lipids, proteins, phosphate groups, and other minor constituents can significantly affect gelatinization-related behavior, film formation, transparency, mechanical strength, flexibility, and barrier properties [67,68,69,70]. After gelatinization, plasticization, drying, or thermal processing, starch can be used to produce continuous polymeric matrices via solvent casting, extrusion, compression molding, or thermoplastic starch (TPS) processing [68,69,70,71]. Cassava and potato starches are widely studied root-derived starches that can yield translucent, elastic films under appropriate formulation and processing conditions, whereas sweet potato starch and pigmented tuber residue can also be considered sources of antioxidant properties or colorimetric attributes [12,67]. Native starch films are generally hydrophilic, sensitive to relative humidity, and brittle; plasticization, polymer blending, reinforcement, and hydrophobic modification are therefore commonly required [68,71,72]. To address these shortcomings, researchers commonly blend these polymers with other components, such as starch with chitosan, gelatin, pectin, polyvinyl alcohol, poly(lactic acid), polybutylene adipate terephthalate, nanocellulose, plant extract, and natural waxes [32].

### 2.2. Biodegradation of Biopolymer Films

The biodegradation of polymeric films involves both abiotic and biological processes. Polymer deterioration can be initiated by UV radiation, temperature, moisture, oxidation, hydrolysis, and mechanical stresses; microbes and enzymes can then break polymer chains into oligomers and monomers. These products are subsequently assimilated and mineralized to CO_2_, water, and microbial biomass under aerobic conditions, whereas anaerobic degradation may generate methane [73,74,75]. Several factors influence the degradation rate, including polymer chemistry, crystallinity, thickness, additives, temperature, moisture, microbial activity, and environmental conditions [13,14,73,74,75].

### 2.3. pH-Responsive Behavior of Natural Pigments

Anthocyanins are naturally occurring pigments that exhibit pH-sensitive molecular equilibria, resulting in a characteristic color change from red in acid to purple/blue at near-neutral pH and greenish yellow at alkaline pH [76,77,78,79]. In this way, they can be used as freshness indicators because pH can change in the surrounding space due to the release of metabolites from microbial spoilage, especially in protein-rich foods, which contain volatile amines. On the other hand, the response of the pigments is influenced by the type of anthocyanins, the polymeric matrix, and moisture, temperature, light, and storage conditions [76,77,78,79,80,81].

## 3. Roots and Tubers as Renewable Sources of Film-Forming Biopolymers

Starch and fructan carbohydrates are film-forming biopolymers of special interest that can be obtained from renewable resources such as roots and tubers. Renewable feedstocks that are rich in starch are among the most significant, such as cassava root, potato, sweet potato, taro, yam, arrowroot, and canna, due to their high nutritional content, availability, biodegradability, and potential for good film-forming properties [66]. Starch is particularly desirable due to its availability, extensive distribution, biodegradability, low cost, and the ability to form thermoplastic materials and continuous films upon gelatinization and plasticization [67,68]. Generally, roots and tubers are promising feedstocks for sustainable polymeric materials because residues generated during processing can be integrated into biorefinery pathways for packaging and agricultural film markets. The application of starch-based materials obtained from roots and tubers in the making of edible films, biodegradable food-packaging materials, coatings for fruits and vegetables, agricultural mulch films, seedling bags, and polymer blends for use in plasticulture has been widely studied. Starch matrices have been plasticized by the addition of glycerol or sorbitol to overcome these limitations, with some concern about the moisture sensitivity of matrices [69,70,71]. Starch is often used in combination with natural and synthetic biodegradable polymers, such as chitosan, gelatin, pectin, poly(vinyl alcohol) (PVA), poly(lactic acid) (PLA), poly(butylene adipate-co-terephthalate) (PBAT), nanocellulose, natural waxes, lipid phases and plant-derived bioactive extracts, to improve its mechanical resistance, water resistance, thermal stability and functional properties [20,72]. These modifications have enabled the development of multifunctional starch-based materials with improved barrier properties, antioxidant and antimicrobial capacities, and intelligent packaging.

Compared with other crops, starch-rich roots and tubers are attractive as low-cost film-forming matrices, although their hydrophilicity and moisture sensitivity in most cases require plasticization, blending, or reinforcement. Filming properties have been demonstrated with cassava and potato, while sweet potato can also provide additional pigment and antioxidant properties. In comparison, topinambur is less suited as the starch matrix of conventional hydrocolloids but offers a unique platform of fructan-based hydrocolloids, along with aerial biomass rich in phenolic compounds and color. Thus, the principal trade-off is between the processing maturity and structural performance of starch-based systems and the broader multifunctionality but lower technological maturity of topinambur-derived materials.

## 4. Biodegradable Films for Agronomic Applications and Plasticulture

In modern intensive farming, plasticulture is utilized globally to control weed growth, conserve soil moisture, regulate soil temperatures, reduce soil erosion, and enhance crop productivity and quality. Polyethylene (PE) remains the predominant film used in agricultural production, but post-cultivation recovery and disposal of these films are incomplete. Persistent PE residues can build up in agricultural soils, may lead to microplastic contamination, and may impact soil quality and agricultural sustainability [32,82,83]. Biodegradable mulch films have therefore been recognized as alternatives that not only provide sufficient field performance during crop production but can also easily break down into environmentally friendly products [84].

Polysaccharides, proteins, biodegradable polyesters, and hybrid bio-composites are suitable materials for producing biodegradable agricultural films (Figure 1). Figure 1 highlights the important trade-off for biodegradable plasticulture: the need for adequate field performance during crop growth and controlled degradation afterward. Using renewable matrices, fibers, nanocellulose, hydrophobic modifiers, and biodegradable polyesters requires balancing durability, renewable content, and biodegradation. Excessive durability can leave persistent residues, while premature breakdown may affect agronomic performance and, consequently, make crop-specific formulation necessary for the crop [73,74,75,85]. Like all mulch films, starch-based films have the general structural and moisture-use constraints described in Section 2.1. They are typically combined with plasticizers, fibers, nanocellulose, waxes, and biodegradable polyesters to improve field performance [67,68,86]. Therefore, plasticizers like glycerol and sorbitol, as well as chitosan, cellulose fibers, nanocellulose, natural waxes, and biodegradable polyesters, are added to increase the flexibility, water resistance, mechanical strength, and field durability of the composite material [67,68,87].

Lignocellulosic biomass plays an important role in biodegradable plasticulture. Cellulose- and fiber-based cellulosic residues from bagasse, rice straw, wheat straw, corn stover, husks, and other agricultural residues are frequently employed as reinforcing materials in the production of biodegradable films. The fiber–polymer matrix interactions can enhance tensile strength, stiffness, dimensional stability, and tear resistance [85,86]. Nanocellulose is especially useful because of its high crystallinity, surface area, and aspect ratio, while lignin components contribute to protection against UV radiation, weed control, and resistance to outdoor environmental damage [83,85,86]. Additionally, biodegradable polyesters, including poly(lactic acid) (PLA), poly(butylene adipate-co-terephthalate) (PBAT), poly(butylene succinate) (PBS), polycaprolactone (PCL) and polyhydroxyalkanoates (PHAs), are employed either alone or in combination with starch and natural fibers [87,88]. PBAT offers flexibility, toughness, and good processing characteristics, whereas PLA exhibits good stiffness and tensile strength but is comparatively brittle. As a result, starch–PBAT and PLA–PBAT blends are considered the most relevant formulations for the production of biodegradable mulch films, since they combine renewable resources with the ductility and processability of PBAT [89].

Furthermore, PHAs are also known to be biodegradable in soil and composting conditions; however, the cost of production and limited mechanical flexibility are still considered major constraints for commercialization [3,8]. For plasticulture, the general degradation processes described in Section 2.2 must be synchronized with crop duration. Premature degradation compromises field performance, whereas excessive persistence after harvest reduces the environmental benefit [72,76,90]. Meta-analyses suggest that the performance of biodegradable mulch films is comparable to PE in some agricultural systems, but results can vary significantly due to crop type, climatic conditions, soil management, film formulation, and mulch management. In view of this, appropriate materials must be selected according to agroclimatic situations [72]. One major unresolved problem is synchronizing film degradation with the crop production cycle. During establishment and growth, mulch films must have adequate tensile strength, UV resistance, and barrier performance and must begin degrading after completing their agronomic function. Premature fragmentation can lead to poor weed control and moisture conservation, while delayed fragmentation can leave residual film material, resulting in inferior environmental benefits compared with polyethylene (PE) [72,76,77,78,91]. The ability of a formulation to degrade is influenced by polymer chemistry, thickness, crystallinity, soil moisture, temperature, exposure to sunlight, and microbial activity, so the same formulation that is effective in some cropping systems or climates may not be equally effective in others. Biodegradable mulch films, therefore, need to be designed with a service life appropriate to a particular crop and region rather than according to a universal degradable target.

Circular bio-economy-aimed approaches can be pursued with the use of biodegradable plasticulture. In this process, low-grade tubers, residues in starch processing, tuber peel fractions, fibers, and by-products from the agro-industrial sector are converted into thermoplastic starch, bio-composites, and molded mulch film. The use of biodegradable polyesters and natural fibers, waxes, nanocellulose, and functional additives can further enhance mechanical, barrier, and weathering properties while increasing the renewable content of the films. However, broader commercialization demands uniform feedstock quality, highly scalable extrusion and film-processing technologies, competitive costs, consistent biodegradation tests, and validation of food and environmental safety, while also necessitating long-term testing under various real-world conditions [92].

The literature indicates that the preparation of biodegradable agricultural films can generally be classified into six technological routes (Table 2): (1) blown-film extrusion: the PHA/PBAT, PLA/PHA, starch/PBAT and PBAT/PPC types are close to the conventional PE film industrial scale-up process and are attractive for commercial scale-up; (2) reactive melt blending + extrusion: these types enable polymer combinations, e.g., the mechanical strength provided by PHA or PBAT is further enhanced by the introduction of ESO (Epoxidized Soybean Oil) or ESA (Epoxidized Succinate/Soybean oil derivative or Epoxidized Fatty Acid), or by the use of PPC (Poly (propylene carbonate)) or PBATP/ADR (Poly (butylene adipate-co-terephthalate) composite/phase)/(Epoxidized chain extender); (3) solution casting/aqueous film formation: these types are suitable for natural polymers and enable the addition of plasticizers, nutrients, bioactive compounds and biomass fillers under relatively mild conditions; (4) spray-on film formation (sodium alginate-based formulations and other water-soluble biopolymers): eliminates the need for laying and later collecting prefabricated sheets, which is particularly interesting for irregular field surfaces and horticulture; (5) hot pressing/multilayer fabrication (PHA/PCL and other multilayer biodegradable combinations): allows individual layers to perform different functions, such as strength, flexibility, moisture barrier and controlled biodegradation; (6) electrospinning/nanocomposite fabrication (PVA/starch, PBAT/cellulose nanofiber, PBAT/lignocellulose nanosheets): enables highly engineered micro- and nanostructures and the incorporation of biomass-derived reinforcing agents, although scale-up remains an important consideration. Overall, biodegradable mulch films represent an advanced application of bio-based and biodegradable polymers in agriculture. Progress in biomass valorization, polymer blending, nanocomposite engineering, and application-specific material design is expected to enable plasticulture films with reliable agronomic performance and predictable end-of-life degradation, thereby reducing agricultural plastic pollution and supporting the transition toward sustainable farming systems.

## 5. Valorization of Yerba Mate By-Products in Biodegradable Composite Development

Yerba mate (*Ilex paraguariensis*) is among the most consumed beverages in South America, and the culture and industrial processing produce a significant amount of spent product, consisting of leaves, stems, dust, and extraction residues (Figure 2). Figure 2 highlights several possibilities for using yerba-mate residues. The lignocellulosic fraction can be used as a structural reinforcement and UV protection, whereas the phenolic fractions can be used for antioxidant and pH-responsive applications. Reinforcement can impact dispersion; however, bioactive extracts change color, moisture sensitivity, and mechanical properties [113,114,115,116,117]. Interestingly, these by-products contain both lignocellulosic and bioactive molecules, such as cellulose, hemicelluloses, lignin, minerals, caffeine, chlorogenic acids, flavonoids, tannins, methylxanthines, and other phenolics, making them advantageous feedstocks for biodegradable composites [113,114,115]. Incorporating them into biodegradable polymer matrices enables their use as reinforcement fillers or functional additives. Results showed that the lignocellulosic fraction can increase the stiffness, dimensional stability, opacity, and UV resistance of starch-, cellulose-, gelatin-, poly(vinyl alcohol) (PVA)-, and biodegradable polyester-based materials [114]. Phenolic-rich extracts exhibit antioxidant properties that can slow lipid oxidation and improve product shelf life. In addition, natural pigment sources and phenolic compounds can produce pH-dependent color changes that can be potentially exploited for the development of intelligent food packaging (Table 3). Additives derived from yerba-mate may also affect hydrophilicity, plasticization, mechanical performance, and barrier performance through interactions with the polymer matrix [116,117].

The versatility of such additives in biodegradable films has recently been proven. Yerba-mate extracts added to starch-based films were produced using extrusion and compression molding techniques and showed enhanced antioxidant activity, altered mechanical properties, developed pH-responsive color, and disintegrated after soil burial [116,117]. The use of microfibrillated cellulose–corn starch films with yerba-mate extract also demonstrated a synergistic effect among the bioactive compounds and the nanocellulosic material, producing improvements in the mechanical performance of the films alongside their antioxidant and antibacterial activity. Industrial yerba-mate residues have also been incorporated into packaging materials, such as cassava starch-based adhesives that promote the valorization of processing residues. Yerba-mate residues are locally available, abundant, and inexpensive raw materials with potential applications ranging from biodegradable coatings and paper composites to agricultural films and other materials. They can be utilized to mitigate waste production and add antioxidant, antimicrobial, UV-protective, and pH-responsive capabilities (Figure 2). However, for commercialization, the extraction process must be standardized, the bioactive composition must remain consistent, and compatibility with the matrix has to be enhanced, along with adaptation to processes of extrusion, compression molding, coating, and lamination [116,117]. Overloading extracts can lead to darkening of films, increased moisture sensitivity, changed odor, or reduced mechanical strength. In addition to the agronomic evaluation, food-contact applications of these materials must be evaluated for migration, toxicological safety, and consumer acceptance. For agronomy, the effects of caffeine, tannins, and phenolics on soil microorganisms and plant growth are still insufficiently understood [113,114,115]. Yerba mate by-products are best considered as multifunctional fillers and bioactive additives rather than stand-alone film-forming materials, capable of enhancing the performance of new-generation biodegradable composites and proposed as supporting principles of circular bioeconomy [118,119]. Unlike starch-based feedstocks, in the proposed framework, yerba mate is recognized as an additional material that does not form the polymer matrix but offers reinforcement and multifunctional bioactivity. The lignocellulosic fraction can have both positive antioxidant properties and possibly pH-dependent properties, while its phenolic component can enhance stiffness and UV-protective properties. However, these advantages depend on formulation; too much extract loading can negatively affect moisture resistance, color, odor, and mechanical performance. Thus, yerba mate is more appropriate as a multifunctional filler or active additive, and it is important to standardize the extract, ensure matrix compatibility, maintain processing stability, and validate safety to industrialize its use as a multifunctional filler or active additive.

**Table 3:** Yerba-mate by-products as multifunctional components in biodegradable composites.

Yerba-Mate Source/By-Product Form	Biodegradable Matrix/Composite	Preparation/Formulation Strategy	Multifunctional Role of Yerba-Mate Component	Main Findings/Potential Application	References
Fine yerba-mate powder generated during industrial processing	Calcium alginate	Yerba-mate powder incorporated into alginate capsules containing urea	Filler + organic matter source + mineral source + release modifier + biodegradation modifier	Yerba-mate powder contents of approximately 30–77% were used in biodegradable alginate capsules. Incorporation of the residue enhanced urea encapsulation and provided a potential biodegradable controlled-release fertilizer system.	[120]
Post-consumption/spent yerba mate infusion residue	PLA/ATBC	Residue was subjected to a solvent-free/hydrothermal process to obtain lignocellulosic mate nanoparticles (mateNP), followed by melt blending with PLA and ATBC	Nanoreinforcement + antioxidant component + thermal stabilizer + UV blocker + crystallization modifier	MateNP improved thermal stability, promoted crystallization, and imparted UV-blocking effects. ATBC improved dispersion and flexibility, while the films disintegrated under composting conditions in approximately one month.	[121]
Yerba-mate biomass valorized into cellulose/nanocellulose and yerba-mate-based extract	Cellulose/nanocellulose-based aerogels	Cellulose and nanocellulose fractions were isolated from yerba mate and used to prepare bioactive aerogel structures containing yerba-mate extract	Structural biopolymer source + nanoreinforcement + carrier matrix + bioactive/antioxidant reservoir	Demonstrated integrated valorization in which yerba-mate biomass can supply both structural cellulose fractions and bioactive compounds for biodegradable delivery materials.	[122]
Lignocellulosic nanoparticles extracted from yerba-mate waste	Recycled PLA/sodium caseinate/recycled PLA multilayer composite	Tri-layer films containing mechanically recycled PLA outer layers and a sodium-caseinate middle layer; 1 or 3 wt% yerba-mate-derived nanoparticles incorporated into the internal PLA layer	Nanoreinforcement + antioxidant agent + oxygen-barrier modifier + waste-derived functional filler	Yerba-mate nanoparticles imparted antioxidant activity and contributed to improved oxygen-barrier performance. The multilayer systems disintegrated under composting conditions within 17 days in the reported test.	[123]
Yerba-mate extract	Cassava starch/glycerol	Yerba-mate extract at different concentrations incorporated into cassava-starch films by solution casting	Antioxidant + plasticizer + hydrophobicity modifier	Incorporation of the extract produced antioxidant films and increased elasticity; changes in surface and moisture-related properties indicated that yerba-mate compounds acted simultaneously as bioactive additives and plasticizing agents.	[124]
Yerba-mate antioxidant extract	Carrageenan/corn starch	Natural extract incorporated into carrageenan–corn-starch films by casting	Antioxidant + controlled-release bioactive + mechanical -property modifier	The films functioned as biodegradable carriers for natural antioxidant compounds. Yerba-mate-containing formulations exhibited relatively homogeneous structures and enabled release of antioxidant compounds into aqueous media.	[125]
Yerba mate extract rich in phenolic compounds/chlorogenic acid	Sodium alginate	Different extract concentrations incorporated into alginate films	Antioxidant + controlled-release agent + mechanical-property modifier	Yerba-mate extract increased tensile strength and Young’s modulus and enabled diffusion-controlled release of total phenolics and chlorogenic acid into food simulants, supporting active antioxidant packaging applications.	[126]
Yerba-mate extract	Carboxymethyl cassava starch/PVA	Starch/PVA blends with glycerol, citric acid and yerba-mate extract processed by extrusion and thermoforming	Antioxidant + compatibilizing/plasticizing contributor + biodegradation enhancer	PVA improved mechanical resistance and hydrophobicity, whereas yerba-mate extract enhanced compost degradation and supplied antioxidant activity. Approximately 90% of extract polyphenols were retained after processing in the reported formulations.	[127]

## 6. Non-Traditional Functional Biomass Sources

### 6.1. Rosehip (Rosa rubiginosa)

Rosehip (*Rosa rubiginosa*) is an underutilized wild fruit and an important source of agro-industrial by-products, with considerable potential for the development of active biodegradable packaging and functional biopolymer composites. Rosehip cultivation and processing for teas, jams, powders, oils, nutraceuticals, and cosmetics generate pomace, peels, and seeds that could be used for biodegradable packaging. The by-products are rich in phenolic acids, flavonoids, carotenoids, tocopherols, unsaturated seed oils, vitamin C, organic acids, and dietary fiber and thus have antioxidant, hydrophobic, and reinforcing functions [128,129,130]. Phenolics, carotenoids, tocopherols, and vitamin C present in rosehips can slow oxidative degradation of lipid-rich foods and prolong shelf life [128,129]. Therefore, rosehip extracts have been used as fillers in polymeric films based on starch, gelatin, chitosan, pectin, and poly(vinyl alcohol) (PVA). Considering that antioxidant activity remains key to food packaging, rosehip extract added to rye starch films protected against lipid oxidation during chicken breast oxidation, whereas the PVA/chitosan films exhibited an increase in antioxidant activity along with alterations in thermal stability, barrier properties, and storage performance in oxidized packaged chicken breast [131,132]. Rosehip seed oil contains high amounts of both unsaturated fatty acids and tocopherols, which can be utilized in emulsion films or coatings as a hydrophobic phase leading to the improvement of water resistance and antioxidant protection, as demonstrated in various studies [128,129]. Fiber-rich pomace and seed residues may also serve as reinforcing fillers, supporting more complete valorization of rosehip processing waste. However, introducing these components into polymer matrices is not always straightforward, as it relies on the compatibility between polymers and formulation conditions. Emulsification or encapsulation may be necessary if the oil phase is required to be dispersed in hydrophilic matrices like starch, gelatin, pectin, chitosan, and PVA, while high extract concentrations may cause changes in color, aroma, microstructure, mechanical properties, migration behavior, and oxidation stability. Therefore, rosehip by-products should be viewed as multifunctional antioxidants, hydrophobic modifiers or reinforcing fillers, and not as separate film-forming materials.

### 6.2. Aloe vera (Aloe barbadensis Miller)

*Aloe vera* (*Aloe barbadensis* Miller) has been extensively researched for use in edible films, coatings, and biodegradable packaging and is considered to be a mixture of film-forming polysaccharides and bioactive compounds [133,134,135]. The gel dry matter is composed of acemannan, a β-(1 → 4)-linked acetylated mannan, glucomannans, pectic substances, organic acids, minerals, amino acids, enzymes, phenolics, and vitamins. *Aloe vera* is rich in the principal hydrocolloid, named acemannan, which is responsible for its hydrocolloid, film-forming, and functional properties [133,134,135]. *Aloe vera* gel has structural and bioactive functions. It can be used to produce transparent, flexible, and edible films or coatings, with the polysaccharides conferring these properties, whilst phenolic and related components exhibit antioxidant and antimicrobial properties. Hence, *Aloe vera* coatings have been employed to control moisture loss, slow respiration, improve mold resistance, and extend the storage time of fruits and vegetables [135,136]. *Aloe vera* is an appealing hydrocolloid for new applications in active food packaging due to its multifunctional properties. However, *Aloe vera* films are generally hydrophilic, with relatively poor mechanical strength and moisture-barrier properties. *Aloe vera* is thus frequently combined with other polymers, such as chitosan, starch, gelatin, alginate, or pectin, to provide greater mechanical strength, thermal stability, barrier characteristics, and functionality [133,137]. Moreover, Aloe vera/chitosan films have exhibited better antioxidant, antimicrobial, thermal, and barrier properties, and chitosan–*Aloe vera* coatings are effective in improving the quality of produce and minimizing postharvest decay caused by diseases [133,137]. *Aloe vera* can be generally regarded as a multifunctional hydrocolloid and bioactive additive rather than as a replacement for other conventional plastics. Other advances in polymer blending, polymer crosslinking, and processing could enhance its potential for future applications in biodegradable composites, active packaging, and edible coatings.

### 6.3. Topinambur (Helianthus tuberosus *L.*)

Topinambur or Jerusalem artichoke (*Helianthus tuberosus* L.) is a promising source of sustainable polymeric materials, since both its fructan-rich tubers and the bioactive aerial biomass can be valorized. Inulin and fructo-oligosaccharides are the predominant carbohydrates that are stored in topinambur, compared to the starch present in other tubers, and are appropriate in hydrocolloid films, edible films and coatings, and edible polymer blends [138,139,140]. The raw material is also rich in dietary fiber, proteins, minerals, organic acids, and phenolic compounds, including chlorogenic acids found in its leaves, flowers, stems, and tubers [140]. The valorization of topinambur is particularly interesting within a circular bioeconomy, since nearly the whole structure of the plant can be valorized. Topinambur is of prime importance because of its high tuber inulin and fructooligosaccharides (fructan-type carbohydrates) content, in which the basic unit is fructose, mainly linked by the β-(2 → 1) glycosidic bond, capped by a glucose unit at the terminal position [138,139]. The properties of inulin, which are suitable for producing biodegradable films, edible coatings, hydrocolloids, and active packaging systems, are water solubility, biodegradability, prebiotic activity, and the formation of hydrocolloids [138]. Inulin content in tubers has been reported to range from 8.16 to 13.46% of fresh weight, depending on genotype, maturity, and growing conditions. Inulin can be blended with a variety of starches, pectins, alginate, gelatin, chitosan, poly(vinyl alcohol) (PVA), and other biodegradable polymers, with hydrogen bonding interactions potentially improving compatibility and functionality [138]. The antioxidant and UV-protective properties of phenolic extracts from leaves and flowers can be used as an active component in the formulation of antioxidant and UV-protective films and coatings [140]. Topinambur flowers have also been proposed as a raw material for the production of natural colorants in intelligent food packaging. Evidence of its pigment composition, pH response, stability, migration, and freshness-monitoring performance remains limited compared with that for established anthocyanin sources, such as red cabbage and berry pomace [140,141]. Generally, topinambur production is more of an opportunity for a complementary multifunctional feedstock than a sole bioplastic resource. Valorization of both tuber and aerial biomass rich in fructans and phenolic compounds affords further substrates for manufacturing biodegradable films, edible coatings, and substrates for novel intelligent packaging applications.

The three non-traditional biomass sources provide complementary rather than equivalent functions. Rosehip extracts, seed oil, and pomace powder are rich in phenolic constituents as well as antioxidants, and their main application is as a hydrophobic modifier, whereas *Aloe vera* is beneficial as a film-forming hydrocolloid and antimicrobial coating agent, but its performance as a film former is limited by its hydrophilicity, which is a major drawback. Topinambur sources the hydrocolloids as fructans and is also rich in phenolic compounds and, in certain applications, even colorimetric compounds or dyes. Thus, rosehip appears more promising for active packaging materials, *Aloe vera* for edible food coatings and blended films, and topinambur for multifunctional films and new intelligent-packaging systems. They share certain drawbacks, including compositional variability, moisture exposure, matrix issues, and a lack of industrial-scale validation (Table 4).

## 7. Active and Intelligent Food Packaging Using Natural Pigments

The active and intelligent packaging market has been one of the fastest-growing industries within the sustainable packaging segment, offering a one-stop shop for food safety, extended shelf life, reduction in food waste, and environmental sustainability. Active packaging interacts with the food or headspace of the package to slow the deterioration process, whereas intelligent packaging detects changes in moisture content, freshness, storage conditions, and even food safety and provides visual or measurable information to producers, retailers, and consumers. Among intelligent packaging technologies, colorimetric pH indicators have been of great interest because many perishable food products experience typical changes in pH during microbial spoilage [79,80,81,142]. Four specific formulation strategies are identified in the representative studies: (i) direct incorporation of natural pigments into biopolymer matrices; (ii) incorporation of pigments with active antimicrobial or antioxidant agents; (iii) pigment stabilization using the technique of nanoencapsulation prior to incorporation into a film; and (iv) advanced formulation techniques, including the incorporation of pigments as indicators in bacterial-cellulose-based films or the preparation of nanofibers containing pigments by electrochemical technology.

Natural pigments are increasingly preferred sensing materials due to their renewability, biodegradability, generally regarded as safe (GRAS) status, and compatibility with bio-based polymer matrices. Based on the pH-response mechanism described in Section 2.3, anthocyanins from red cabbage, purple sweet potato, berry residues, and other biomass sources have been incorporated into biodegradable matrices based on starch, chitosan, gelatin, pectin, alginate, cellulose derivatives, agar, carrageenan, or poly(vinyl alcohol) (PVA) for freshness monitoring [50,82,83,84,143,144,145,146,147]. Similarly, curcumin-containing films are sensitive to alkaline pH and ammonia, while beetroot extracts rich in betalains and extracts from butterfly pea flowers have been identified as promising alternative sensing films [66]. The details of intelligent food packaging using natural pigments and their mechanisms, with different examples, are illustrated in Figure 3. Figure 3 shows a schematic representation of the connection between natural pigments, polymer matrices, the development of food spoilage, and visual freshness monitoring. When proteins start decomposing, volatile amines are generated that cause color changes in anthocyanin-type indicators as a function of pH. The properties of their performance are governed by pigment chemistry, matrix properties, fabrication format, and their application, which relies on sensitivity and stability, migration control, and food system compatibility [50,79,80,81,82,83,84,142,143,144,145,146,147].

The growing availability of natural pigments recovered from agro-industrial by-products further strengthens the sustainability of intelligent packaging. Pigments obtained from red cabbage waste, grape pomace, berry pomace, purple sweet potato peel, and flower residues are not only cost-saving given raw material availability but can also support circular bioeconomy strategies by turning agricultural wastes into value-added products from different streams [50]. In this context, topinambur (*Helianthus tuberosus*) flowers have recently captured interest as a possible renewable source of pigments. Based on preliminary studies, flower extracts are reported to have antioxidant properties and colorimetric potential. Still, systematic investigations into pigment composition, extraction efficiency, pH-response characteristics, light and heat stability, pigment migration, and interactions with biopolymer binders during real food storage are yet to be conducted. Therefore, topinambur flower pigments should still be considered a potential supplement, rather than a substitute, for existing sources like red cabbage [39]. Representative examples of natural pigment-based active and intelligent food-packaging systems are presented in Table 5. Overall, active and intelligent packaging based on natural pigments represents one of the most promising directions in sustainable food packaging. Continued integration of biodegradable polymers, agro-industrial by-product valorization, advanced pigment stabilization technologies, and scalable printing and coating processes is expected to accelerate the commercialization of environmentally friendly packaging systems capable of enhancing food safety, reducing waste, and supporting the transition toward a circular bioeconomy.

### 7.1. Limitations of Anthocyanin-Based Indicators

Anthocyanin indicators have high pH sensitivity but are relatively unstable under photothermal stress and during storage. The indicators will lose their reliability if pigments are degraded, faded, or permanently shifted by light, oxygen, high temperatures, moisture, and/or long storage. Furthermore, weak interactions with highly hydrophilic polymer matrices may allow pigment ‘diffusion’ or ‘migration’, especially at high moisture content, which could result in a loss of sensitivity and food-contact concerns. The performance of indicators is therefore strongly related to these factors and the environment within the packaging, and these factors are in turn related to matrix properties, interactions between the matrix and indicator, and water activity [50,82,83,84,143,144,145]. Response rate may decrease and production complexity increase with encapsulations, polymer complexation, multilayer immobilization, and UV-protective barriers, but these approaches can enhance stability [143,148].

### 7.2. Sensing Mechanisms and Target Spoilage Signals

In contrast, intelligent packaging differs from active packaging by its ability to detect changes in the status of the food and/or package and provide information about food status. In natural-pigment-based systems, primary responses occur in response to changes in pH and volatile metabolites induced by the process of spoilage. Microbial degradation and enzymatic reactions in protein-rich foods produce volatile basic compounds such as ammonia and amines, which change the microenvironment inside the package and cause an observable reaction in a pH-sensitive indicator. The performance of the indicator is therefore a function of the analyte generation, transportation through the package headspace or the matrix interaction with the sensing compound, and conversion of this interaction into a measurable color signal [50,79,80,81,82,83,84,142].

### 7.3. Natural-Pigment-Based Intelligent Indicators

Anthocyanins are the most studied natural indicators, as their molecular structure provides excellent color change over a wide pH range and they can be bound to starch, chitosan, gelatin, cellulose, alginate, and related biodegradable matrices [50,82,83,84,143,144,145,146,147]. For this reason, red cabbage, purple sweet potato, berries, grape residues, and other anthocyanin-rich biomass sources have been explored for freshness monitoring of fish, shrimp, meat, and poultry. However, intelligent packaging is not restricted to anthocyanins. Curcumin-based indicators are highly sensitive to alkaline conditions and ammonia; betalains uniquely respond to pH; and chlorophyll-related pigments are highly pH-sensitive and exhibit color changes under acidic conditions [13,66,141,143,144]. These pigments vary in reversibility, stability, matrix compatibility, and response range across different spoilage environments. Thus, pigment selection must match the characteristics of metabolites and the pH changes that occur naturally in the target food rather than being based solely on color intensity.

### 7.4. Matrix Engineering and Signal-Regulation Strategies

The polymer matrix and immobilization process of natural pigments strongly affect their sensing performance. Direct incorporation is straightforward but may allow pigment migration within or from the polymer matrix and expose the indicator to light, oxygen, heat, and moisture. Color stability and color retention may be enhanced by copigmentation, adsorption, encapsulation, nanocomplex formation, electrospinning, multilayer immobilization, and incorporation of nanocellulose or other functional carriers [143,148]. For instance, nanoencapsulated anthocyanins have been shown to have higher pigment stability and moisture resistance than free anthocyanins, but they can sometimes require more complex processing or exhibit different response kinetics [148]. These strategies create trade-offs among indicator sensitivity, response time, color contrast, stability, migration resistance, and manufacturability that must be optimized during indicator design. Sensing compounds throughout an entire packaging film may be impractical for commercial use because labels, pads, coatings, and printed indicators can be produced at more commercially viable costs and can separate sensing from structural functions.

### 7.5. Commercial and Practical Constraints

Although much development work has been carried out in the laboratory, challenges remain in translating natural-pigment intelligent packaging into commercial products. Baseline color drift and sensitivity loss, due to pigment degradation under light, oxygen, temperature, and humidity, may threaten reliability and food-contact safety due to migration and interactions between the pigment and food components. Other factors, such as food composition, headspace, storage temperature, microbial ecology, and spoilage pathways, also make universal calibration challenging. Commercial systems, therefore, must have standardized response thresholds, reproducible color interpretation, migration and toxicological evaluation, scalable production, and validation under realistic storage and distribution conditions. For future development of the indicator, special attention should be paid to stabilizing labels and pads, controlling pigment immobilization, multicomponent sensing, and calibration with a smartphone using application-specific calibration. The primary challenge is no longer merely to show the color change in a natural pigment but to ensure that the response is stable, reproducible, food-specific, safe, and economically viable for large-scale production.

## 8. Processing and Formulation Strategies

Integrated processing and formulation strategies are needed to transform agro-industrial by-products, roots, tubers, and natural pigments into biodegradable films. Material performance depends not only on the chemical composition of the feedstock but also on the effectiveness of chemical pretreatment, extraction type, the use of a polymer co-monomer, plasticization, the film-forming process, drying conditions, composite architecture, and post-processing stabilization treatment. Based on this, the evolution of biodegradable and intelligent films should be designed as a series of intertwined steps from raw-material preparation to biopolymer/bioactive extraction, film production, composite design, functionalization, and performance validation (Figure 4). As summarized in Figure 4, the integrated pathway from biomass selection to end-of-life management also encompasses stability, functionality, scalability, and biodegradation, all of which depend on the path the biomass takes from production to extraction, formulation, processing, and, finally, material architecture and management. The figure then relates biomass characteristics and processing strategies to the application-specific performance requirements detailed throughout the review.

### 8.1. Raw-Material Preparation and Extraction

Agro-industrial by-products contain moisture, sugars, fiber, protein, pigments, phenolics, oils, and minerals, which vary by product and require sorting, washing, drying, milling, sieving, and controlled storage. The drying process should be optimized to reduce losses from microbial and enzymatic degradation while preserving thermolabile compounds such as anthocyanins, vitamin C, phenolics, and essential oils [13,141]. Starch from roots and tubers is usually processed by peeling, grinding, water extraction, filtering, sedimentation or centrifugation, drying, and milling to obtain starch, followed by gelatinization and plasticization during film-formation. Cellulose from fiber-rich residues can be recovered by alkaline treatment, bleaching, mechanical fibrillation, or acid hydrolysis, and the resulting cellulose, microfibrillated cellulose, or nanocellulose can be used to improve the mechanical and barrier properties [151,152]. Bioactive compounds and pigments are generally extracted using water, hydroethanolic solvents, acidified hydroethanolic solution/solvent, and ethanol. Mildly acidic conditions are preferred for anthocyanin recovery [13,141], and conventional solvent extraction, as well as other methods such as ultrasound, microwaves, and pressurized liquid extraction [13], can be used to obtain phenolic-rich extracts. Food-grade and green solvents should be preferred to improve safety and sustainability.

### 8.2. Film Production Methods

The principal methods of production of the biodegradable films include solvent casting, extrusion, compression molding, coating, and printing [11,67]. Solvent casting can be used for investigations and for heat-sensitive additives; starch gelatinization acts as the matrix, and improved flexibility is provided by the use of a glycerol or sorbitol solution [11,67]. However, solvent casting can be difficult to scale for industrial production.

Extrusion is a continuous, industrially relevant process in which biopolymers, plasticizers, and fillers, as well as bioactive compounds, can be processed into films by compression molding, cast-film, or blown-film extrusion [11]. Yerba-mate extract has also been used in extruded starch films [116]. The blown-film technique offers specific applications for mulch films like PBAT-, PLA-, thermoplastic starch- and starch–PBAT-based films [76]. Labels, pads, paper, and multilayer packages can be coated and printed with pH-sensitive layers [13,141].

### 8.3. Composite Design

Since single-component biopolymers often fail to fulfill all performance requirements, they are frequently composed of blends, reinforcements, compatibilizations, or multilayers [11,12]. Starch–PBAT, starch–PLA, and PLA–PBAT blends provide a balance of renewable materials, flexibility, and strength, with the inclusion of compatibilizers frequently required to create good interfacial adhesion [76].

Cellulose, lignocellulosic fibers, and nanocellulose can improve strength, stiffness, and thermal stability, but excessive loading can cause aggregation and make the material brittle [151,152]. Moisture resistance is enhanced by the incorporation of waxes, lipids, and hydrophobic polyesters, while problems in dispersion can negatively affect the matrix [11,12]. In multilayer systems, structural, barrier, active, and degradable properties are separated; for instance, an outer layer of polyester for physical durability and an inner layer of starch for degradation [11,67].

### 8.4. Linkage with Bioactive Compounds

Biodegradable films can be functionalized with phenolics, flavonoids, tannins, essential oils, carotenoids, tocopherols, and anthocyanins to impart antioxidant, antimicrobial, UV-protective, oxygen-scavenging, ethylene-modulating, and spoilage-indicating properties [11,141]. Rosehip extracts provide antioxidant and coating functions and also have a protective effect against lipids and microorganisms [132,133], and antioxidant and pH-responsive activity is offered by extracts derived from yerba mate [116,117].

Performance depends on their interaction with the polymer matrix. Phenolic compounds can interact with starch, gelatin, chitosan, cellulose, or PVA via hydrogen bonding, forming stable films, while essential oils can form hydrophobic domains to enhance moisture barrier and antimicrobial properties but can reduce film-forming stability in certain cases. Hydrogen bonding, electrostatic interaction, and physical entrapment are the main mechanisms for immobilizing anthocyanins [13,141]. To increase stability and control release, a variety of physical methods, such as encapsulation, nanoemulsions, cyclodextrins, liposomes, protein–polysaccharide complexes, and multilayer structures, can be used [13].

### 8.5. Intelligent Packaging Formulation

Natural-pigment indicators need to be stable in storage and sensitive to spoilage. Anthocyanins are used extensively due to pH-dependent color changes ranging from red to purple and from blue to green and yellow. Anthocyanins from red cabbage have been entrapped in chitosan, starch, cellulose acetate, and PVA matrices to monitor freshness quality in fish, shrimp, meat, and seafood [82,141].

Typically, formulation includes extraction, stabilization, immobilization, and validation of the pigment with real foods. The matrix selection determines sensitivity and stability: chitosan has antimicrobial properties; starch and PVA are both good transparent carriers, whilst cellulose and paper can be used in labels, and gelatin, agar, carrageenan, and alginate are biodegradable or edible systems [13,141]. Compared to the full intelligent film, indicator labels, smart pads, and printed inks are more commercially viable, as they require less pigment, lower cost, lower risk of migration, and less complexity when adhering to regulations [82,141].

### 8.6. Key Formulation Considerations

Formulation should be tailored to the intended application. Food-packaging films should exhibit high strength, flexibility, barrier performance, safety, and scalability; plasticulture films should resist degradation in the field but also should degrade in a controlled manner; and intelligent systems should remain stable throughout their intended service life while allowing appropriate end-of-life degradation, low component migration, and adequate barrier performance [76,141]. The selection of feedstock, additives, processing, and end-of-life route should therefore be chosen based on the intended use.

### 8.7. Cross-Route Technical Trade-Offs

Processing routes have different principal bottlenecks. Polysaccharide-rich films are renewable and rapidly biodegradable but moisture-sensitive, while polyester blends generally provide higher mechanical strength, although their field durability may still be insufficient under some conditions; lignocelluloses impart high stiffness and barrier properties but may be prone to aggregation and interfacial defects when loaded. Natural-pigment indicators are environmentally benign and low-cost but photothermally unstable and prone to migration. Hence, formulation optimization should aim to achieve both functional properties during use and ease of degradation at end-of-life, rather than maximizing a single material property.

## 9. Characterization Requirements

By-product-based films should be evaluated through complementary mechanical, barrier, structural, thermal, functional, safety, and biodegradation analyses. Mechanical tests include tensile strength, elongation, modulus, puncture, tear, and sealing performance, while agronomic films also require machine-laying and field-durability assessment. Barrier characterization should cover water-vapor and gas permeability, moisture sorption, swelling, solubility, and oil resistance. Structural and thermal analyses commonly include FTIR, SEM, XRD, DSC, TGA, and colorimetry to assess molecular interactions, morphology, crystallinity, thermal stability, and pigment behavior (Table 6).

Functional evaluation should verify antioxidant, antimicrobial, UV-blocking, pH-responsive, color-stability, release, and real-food monitoring performance. Biodegradation should be assessed through soil burial, composting, enzymatic degradation, mass loss, molecular-weight reduction, mineralization, and ecotoxicity. Field trials remain essential for agricultural films because laboratory tests do not fully reproduce actual environmental conditions [141,142,153].

**Table 6:** Analytical characterization for the determination of the physicochemical properties of produced biopolymers [88,108,111,125,141,152,154].

Property Category	Parameters	Suitable Techniques
Physical properties	Density, porosity, swelling, water uptake, thickness	Density test, swelling test, contact angle, porosity analysis
Morphology	Surface structure, cracks, pores, filler dispersion	SEM, TEM, AFM, optical microscopy, micro-CT
Chemical structure	Functional groups, bonding, molecular changes	FTIR, Raman, NMR, XPS
Crystallinity	Crystal structure, amorphous/crystalline ratio	XRD, DSC
Mechanical properties	Tensile strength, modulus, elongation, compression, hardness	UTM, tensile test, compression test, DMA, nanoindentation
Thermal properties	Tg, Tm, thermal stability, degradation temperature	DSC, TGA, DMA, TMA
Functional properties	Antibacterial, antioxidant, barrier, conductivity, controlled release/bioactive-compound release	UV–Vis, zone-of-inhibition, DPPH, WVTR, OTR, impedance
Environmental/biological compatibility	Plant compatibility, soil-microbial effects, migration and ecotoxicological response	Plant-germination/growth tests, soil-microbial assessment, migration and ecotoxicity assays
Biodegradation	Weight loss, CO_2_ evolution, pH change, molecular weight loss	Soil burial, composting, enzymatic test, GPC, SEM, FTIR, respirometry

## 10. Research Gaps and Future Perspectives

Despite this promising progress, there remain some scientific and technical challenges that need to be addressed to industrialize on a large scale. Substantial variability in plant species, geographical and climatic conditions, harvest time, and processing history can cause significant variation in the properties of the feedstocks. The main challenges consist of moisture sensitivity, control of specific biodegradation, pigment instability, feedstock variability, and insufficient validation under real use. The challenge for starch-, pectin-, inulin-, and *Aloe vera*-based films is to make them water-resistant while maintaining their biodegradability. Agricultural film should be evaluated for degradation according to the crop cycles and soil conditions, while packaging film should be tested under realistic composting and waste-management systems.

The pH responsiveness, antimicrobial, and antioxidant performance should be verified not only in model solutions but in real packaged foods. Anthocyanins of red cabbage should be better protected against reactions to light, heat, oxygen, or pH drift, and the pigments of the topinambur flower should be chemically and functionally characterized. Feedstock standardization is also important, as there is a huge variability in composition depending on the cultivar, season, processing, storage, and geographical region.

Issues related to migration, sensory changes, food-contact safety, soil ecotoxicity, and microbial effects should be addressed in future studies. Extrusion, coating, printing, field testing, pilot production, techno-economic analysis, and life-cycle assessment should be emphasized to enable industrial adoption. Thus, ongoing research and development efforts should focus on standardized feedstock characterization, development of green fractionation processes, the use of nanostructured reinforcement materials, multifunctional composite design, and artificial intelligence-assisted optimization of formulations to promote the commercialization of agro-industrial residue-based polymeric materials and to facilitate their transfer from the laboratory to industrial-scale production.

For commercial translation, technical performance, economic feasibility, environmental benefit, and regulatory compliance must be achieved. Feedstock availability, process efficiency, use of solvents and energy, product consistency, compatibility, and end-of-life management are some of the key concerns. Successful scale-up requires pilot-scale process validation and verification of product quality, including by extrusion, compression molding, film blowing, coating, and printing. Techno-economic analysis and life-cycle assessment are necessary to determine whether biodegradable materials are commercially viable and considered environmentally preferable.

### Quantified Research Gaps and Future Perspectives

One remaining major drawback of polysaccharide films is their moisture sensitivity, the degree of which varies considerably with conditions of temperature and humidity in the surrounding environment. For instance, the tensile strength of potato-starch film was reported to decrease from approximately 5.33 MPa at 50% relative humidity (RH) to 0.84 MPa at 75% RH, as reported by Wu et al. [155]. Hence, the corresponding tensile strengths increased to 11.76 MPa (50% RH) and 3.36 MPa (75% RH), respectively, by incorporating 15% agar, which has shown that reinforcement is capable of mitigating, but not eliminating, the mechanical deterioration caused by humidity. Similarly, Lawton [156] demonstrated progressive reduction of ultimate tensile strength with increasing RH (15–93%) in starch/PVA, with elongation increasing due to moisture, which was thought to act as a plasticizer. Based on these results, the use of other starches, pectin, inulin, and *Aloe vera* derivatives as materials should be tested under application-relevant RH conditions rather than applying only standard laboratory conditions.

Instability of pigments should also be numerically assessed as well as expressed in general. Anthocyanins are light-sensitive compounds. Jia et al. investigated blackcurrant (*Ribes nigrum* L.) extract and found that subjecting it to first-order degradation resulted in estimated half-lives of ~8.25 days under natural light and 18.81 days under artificial light, while degradation in dark conditions produced a half-life of ~21 days [157]. This significant difference makes it important to avoid uncontrolled illumination because it can cause pigment loss and baseline color drift and can make indicators even less reliable. The recent literature has strongly highlighted pigment stability in relation to the influence of light, pH, temperature, oxygen, and anthocyanin structure [158]. Future intelligent-packaging studies should report pigment retention, color difference (ΔE), response sensitivity, degradation kinetics under controlled light, temperature, oxygen, and humidity, and appropriate dark-storage evaluations.

Biodegradable agricultural films should be evaluated for degradation during a defined crop-service period rather than just reported as percent mass loss. The experimental field life for mulch films biodegraded by nearly 90% was 21–58 months, longer than the laboratory-tested 24 months set in EN 17033 for field conditions [159]. Sintim et al. further showed a high degree of climate dependency, with the biodegradable mulch films losing a significant percentage of surface area, about 61–83%, after 36 months in Tennessee, while the percentage of degradation in the cooler soil of Washington was much less (26–63%) over the same exposure period. In compost, degradation ranged from 85 to 99% after 18 weeks of exposure, whereas after 36 months in soil, degradation reached 61% to 83% in Knoxville and 26% to 63% in Mount Vernon [160]. The data indicate that this controlled-condition biodegradability is not directly related to persistence in the agricultural field. Future studies should therefore report not only the hours of functional integrity during growing periods, but also the hours of degradation under representative soil and climate conditions, moisture, and cropping conditions. Crop- and region-specific degradation windows could provide a more meaningful design criterion than universal lab degradation targets.

## 11. Conclusions

This review illustrates the potential of agro-industrial by-products and underutilized biomass to be used not only as cheap fillers from waste but also as multifunctional materials for biodegradable polymer matrices, reinforcing phases, bioactive additives, and natural sensing compounds. Despite their many attractive properties as film-forming feedstocks (availability, ease of biodegradation, ability to use existing casting and thermoplastic processing routes), moisture sensitivity and limited wet mechanical performance are important limitations for starch-rich roots and tubers like cassava, potato, and sweet potato. While Topinambur offers an interesting alternative with fructans as the major carbohydrate components, the material’s amount and composition restrict its use as the sole film-forming component; in contrast, yerba-mate residues are better suited as reinforcing, antioxidant, and UV-protective components of food films as well as functional ingredients, rather than as stand-alone film-forming materials. Rosehip and *Aloe vera* also serve specialized antioxidant, hydrophobic, antimicrobial, and hydrocolloid roles, which are most useful in combination with other ingredients in composite or blended products. However, commercial deployment requires improvements in moisture resistance, mechanical performance, pigment stability, and controlled biodegradation, together with comprehensive safety assessment.

In food-packaging applications, the evidence reviewed suggests that composite designs, rather than a single biopolymer, are often necessary to achieve multifunctionality. Blending, fiber or nanocellulose reinforcement, hydrophobic modification, multilayer structures, and incorporation of plant-derived bioactives can enhance mechanical, barrier, antioxidant, and antimicrobial properties. However, these improvements may be limited by moisture sensitivity, dispersion, matrix compatibility, color, migration, and processing complexity. Anthocyanin-based indicators are among the most advanced natural-pigment-based systems, having been validated in a number of perishable food products and offering a wide range of pH-responsive color changes. However, pigment photothermal instability, pigment migration, response reproducibility, and calibration under real storage conditions remain unsolved. Other natural pigments and new sources like Topinambur flowers enhance sensing possibilities, but they have far less technological maturity overall and need further validation for chemical stability, migration, and real-food validation requirements.

Among biodegradable plasticulture options, starch–polyester blends and biodegradable polymer systems based on PLA, PBAT, PHA, alginate, or lignocellulosic reinforcement show strong potential to reduce the use of traditional polyethylene mulch. However, their applicability will rely on a crop-specific balance between the mechanical properties they offer during cultivation and their degradation rate after cultivation/field use. The biodegradability of the film does not necessarily translate to field performance, as it depends on many factors, including formulation, film thickness, soil temperature, moisture, UV exposure, microbial activity, and crop duration. In fact, the key challenge in commercializing all application areas is combining mechanical and barrier performance, moisture resistance, functional stability, controlled biodegradation, feedstock consistency, food and soil-safety requirements, regulatory compliance, competitive processing costs, and industrial scalability. Future research should therefore highlight standardized feedstock characterization, pilot-scale extrusion, coating/printing development, real-food and field validation, migration and ecotoxicity assessment, and techno-economic and life-cycle assessment. The most promising way forward is application-specific: designing multifunctional biopolymer systems with agro-industrial residues that are selected and treated according to their structural, functional, and end-of-life requirements in a circular bioeconomy.

## Figures and Tables

**Figure 1:**
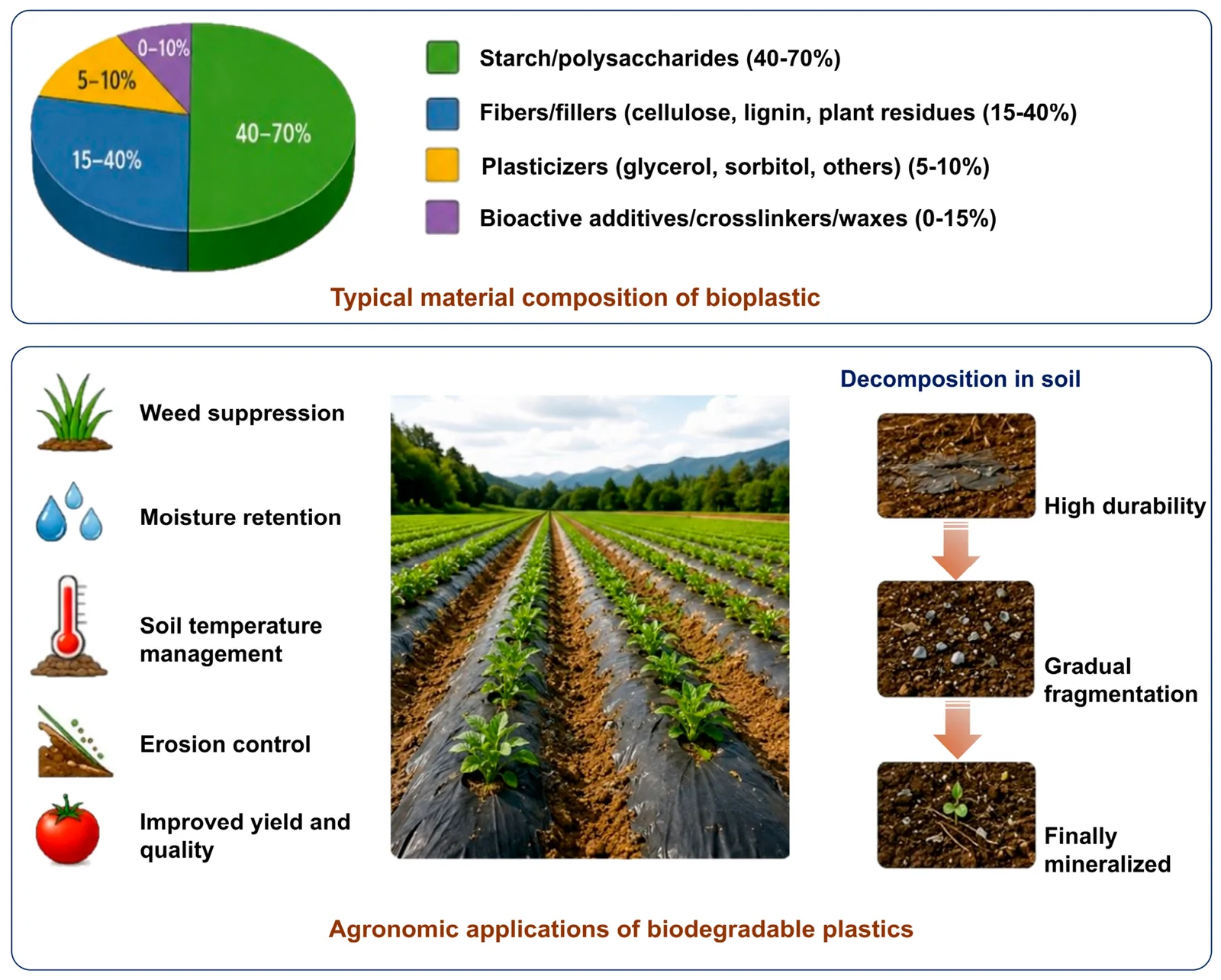
A typical material composition of a biodegradable plastic sheet obtained fromagro–waste and its agronomic applications, including moisture retention, weed suppression, soil–temperature regulation, erosion control, and improved crop yield, along with its subsequent decomposition and mineralization in soil.

**Figure 2:**
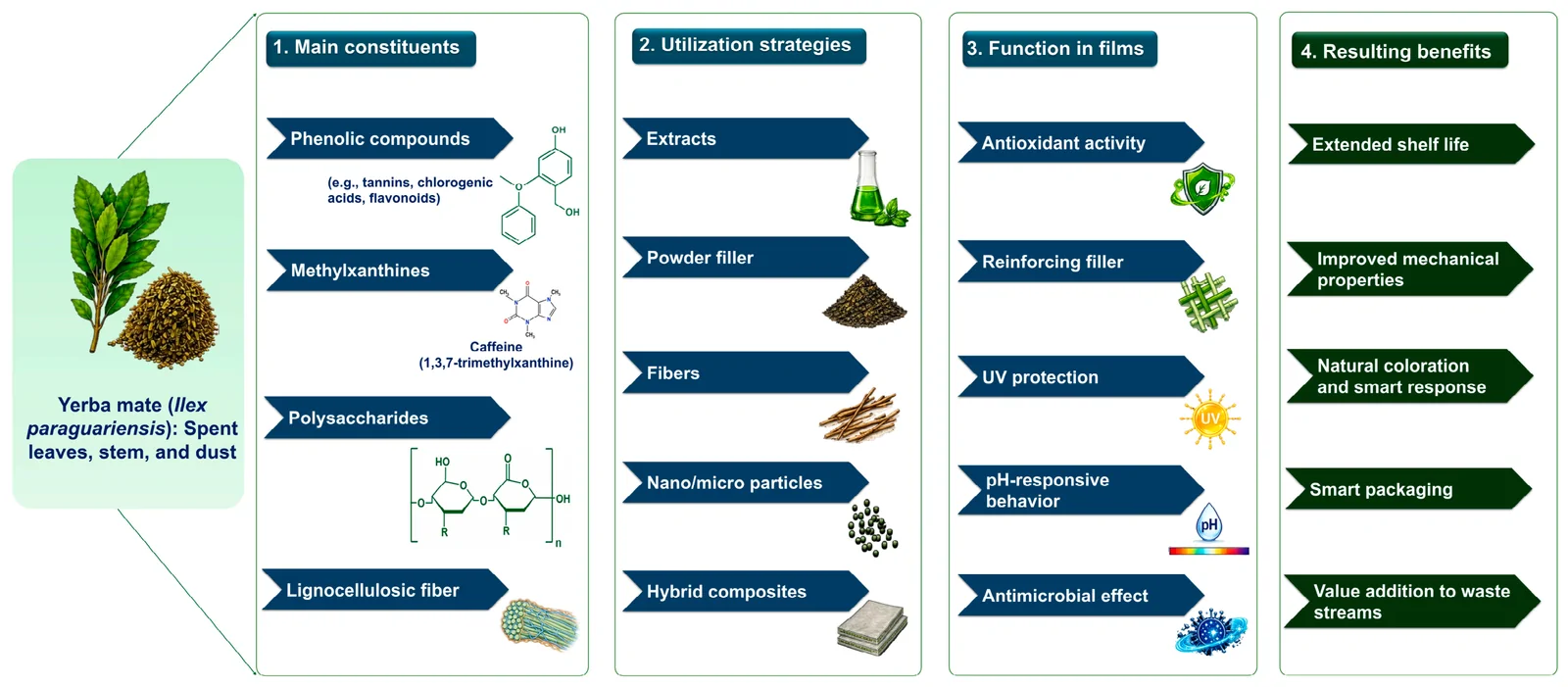
Valorization pathways for spent yerba-mate (*Ilex paraguariensis*) parts (e.g., principal bioactive and other constituents) through the utilization of extracts, fillers, fibers, nano/microparticles, and hybrid composites, the corresponding applications in biodegradable films, and the resulting benefits for smart packaging manufacturers.

**Figure 3:**
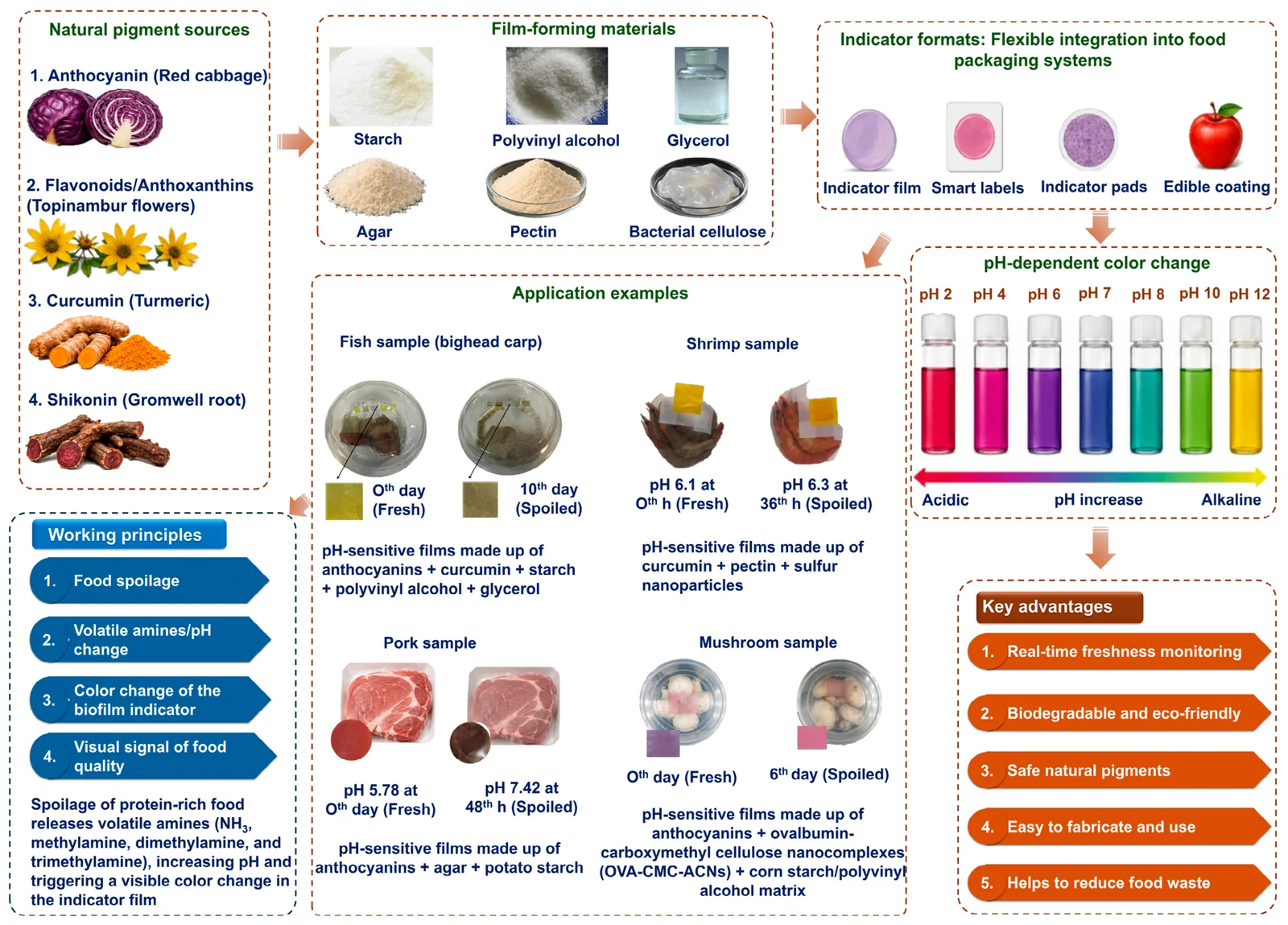
Schematic representation of an overview of pH-responsive intelligent food packaging indicators fabricated from natural pigments and biodegradable plastic materials. It illustrates fabrication formats, pH-dependent color changes, and a spoilage-detection mechanism. It represents potential applications in fish, shrimp, pork, and mushroom monitoring (based on previous studies reported by [79,81,83,139]) as well as the main advantages for real-time food-freshness assessments.

**Figure 4:**
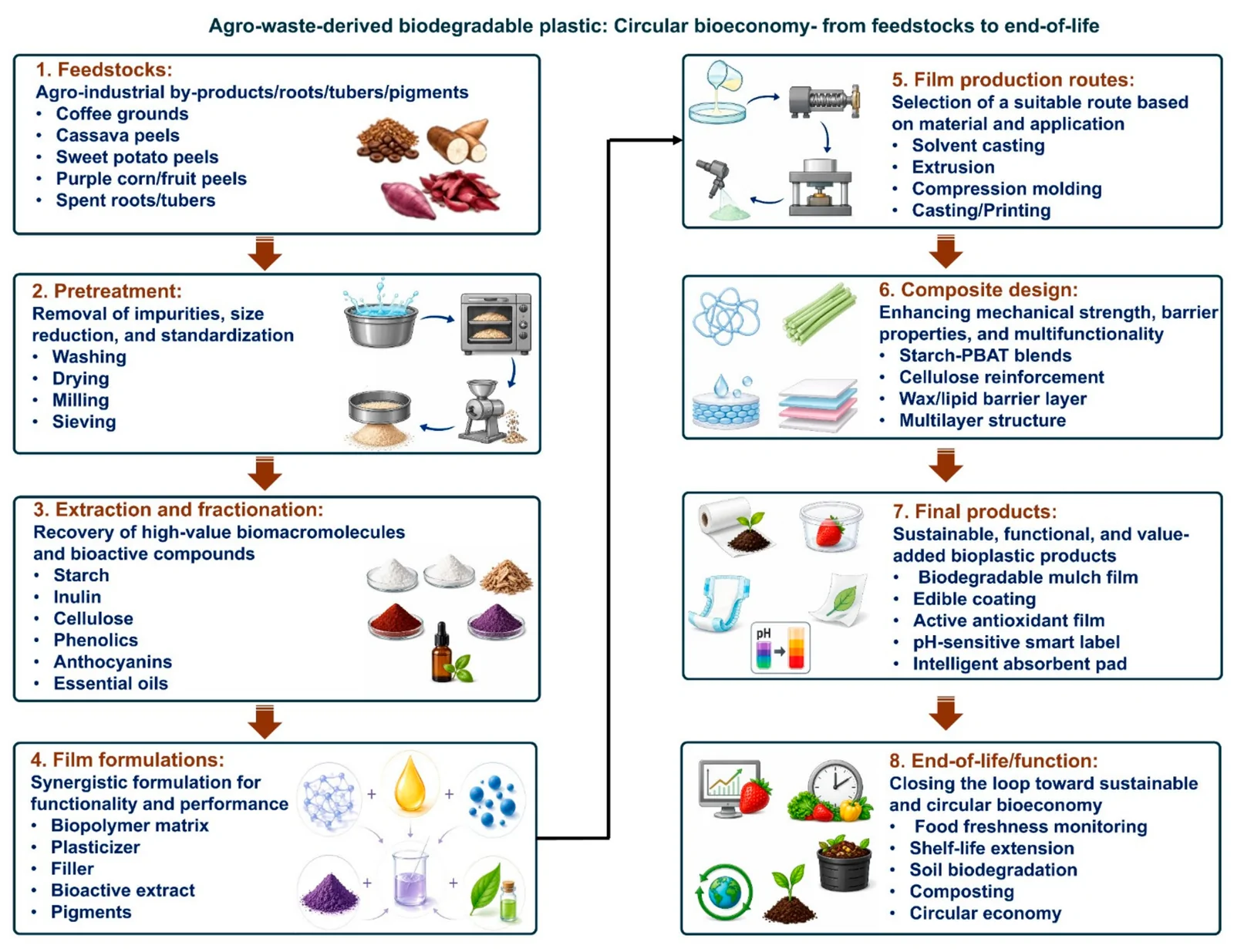
Schematic of integrated production pathway for agro-waste-derived biodegradable films. It shows feedstock selection, extraction and fractionation of functional biocomponents, film formulation and processing, composite design, final product development, and end-of-life functions within a circular bioeconomy framework.

**Table 1:** Agro-industrial by-products as an emerging source of biodegradable polymers, reinforcing fillers, bioactive components, and smart-packaging ingredients.

Agro-Industrial By-Product	Major Useful Compounds	Role in Biodegradable Materials	Potential Applications	References
Potato peel	Starch, cellulose, phenolics, dietary fiber	Film-forming matrix, reinforcing filler, antioxidant additive	Food packaging films, agricultural mulch films	[41,42,43,44]
Cassava peel	Starch, cellulose, hemicellulose, lignin	Starch-based matrix, fiber reinforcement	Bioplastic films, plasticulture films	[43,44,45]
Sweet potato peel	Starch, anthocyanins, carotenoids, dietary fiber	Matrix polymer, natural pigment, antioxidant filler	Active and intelligent packaging films	[43,44,46]
Taro peel	Starch, mucilage, dietary fiber	Hydrocolloid matrix, reinforcing filler	Edible films, biodegradable coatings	[44,47]
Yam peel	Starch, mucilage, phenolics, fiber	Matrix polymer, reinforcing filler	Packaging films, agricultural films	[44,47]
Jerusalem artichoke (topinambur) tuber residues	Inulin, fructans, dietary fiber, phenolic acids	Carbohydrate matrix, functional additive	Active films, biodegradable coatings, polymer blends	[45,48,49]
Jerusalem artichoke (topinambur) flowers	Flavonoids, phenolics, natural pigments	Natural colorant, pH-responsive indicator	Intelligent packaging labels and freshness indicators	[48,49,50]
Yerba-mate spent leaves	Chlorogenic acids, flavonoids, tannins, methylxanthines, lignocellulosic fibers	Antioxidant extract, reinforcing filler, UV-blocking and pH-responsive additive	Active and intelligent food packaging	[51,52]
Tea processing waste	Catechins, tannins, cellulose, lignin	Antioxidant filler, UV-protective additive	Active packaging films, agricultural mulch composites	[53,54,55]
Spent coffee grounds	Cellulose, hemicellulose, lignin, lipids, phenolics	Reinforcing filler, hydrophobic modifier, antioxidant additive	Composite films, trays, biodegradable foams	[53,54,55]
Mixed fruit pomace	Pectin, cellulose, phenolics, anthocyanins	Pectin matrix, reinforcing filler, antioxidant additive	Edible films, active packaging	[53,54,55,56,57]
Apple pomace	Pectin, cellulose, polyphenols	Film-forming matrix, antioxidant additive	Edible coatings, biodegradable packaging films	[53,54]
Citrus peel	Pectin, essential oils, flavonoids, cellulose	Matrix polymer, antimicrobial additive, aroma-active component	Active packaging, edible coatings	[53,54]
Grape pomace	Anthocyanins, tannins, cellulose, grape seed oil	Antioxidant filler, natural colorant, freshness indicator	Active and intelligent packaging	[53,54]
Pomegranate peel	Tannins, ellagic acid, phenolics, cellulose	Antioxidant and antimicrobial additive	Active food packaging	[53,54,58]
Mango peel	Pectin, carotenoids, phenolics, cellulose	Film-forming matrix, antioxidant additive	Edible films, active packaging	[59]
Banana peel	Starch, cellulose, pectin, phenolics	Reinforcing filler, matrix modifier, antioxidant additive	Biodegradable films and composites	[60]
Pineapple peel/core	Cellulose, hemicellulose, bromelain, phenolics	Fiber reinforcement, bioactive additive	Reinforced films, active packaging	[53,54,61]
Sugarcane bagasse	Cellulose, hemicellulose, lignin	Reinforcing fiber, nanocellulose source	Agricultural mulch films, trays, biocomposites	[62,63,64]
Rice husk	Silica, cellulose, lignin	Reinforcing filler, barrier enhancer, stiffness modifier	Composite films, rigid biodegradable packaging	[62,63,64]
Wheat straw	Cellulose, hemicellulose, lignin	Fiber reinforcement, nanocellulose precursor	Agricultural films, packaging composites	[62,63,64]
Corn husk/stover	Cellulose, hemicellulose, lignin	Fiber reinforcement, reinforcing filler	Plasticulture films, molded packaging	[62,63,64]
Coconut husk/coir	Cellulose, lignin-rich fibers	Reinforcing fiber, moisture-resistant filler	Biocomposites, trays, agricultural mulch sheets	[65]
Soybean hull	Cellulose, hemicellulose, proteins	Reinforcing filler, protein–polysaccharide blend	Biodegradable films and coatings	[65,66]
Peanut shell	Cellulose, lignin, phenolics	Reinforcing filler, biochar precursor	Composite films, trays, mulch materials	[53,54]

**Table 2:** Review of the literature on preparation of biodegradable films for agronomic applications and plasticulture.

S. No.	Biodegradable Film/Material	Preparation/Fabrication Method	Modification/Additives Used	Key Properties and Agronomic Relevance	References
1	Sodium alginate-based film	A water-based sodium alginate formulation was developed as a sprayable mulch coating that could be applied directly onto the soil rather than laid as a conventional prefabricated plastic film.	Sodium alginate, a polysaccharide derived from brown seaweed, served as the principal film-forming matrix.	The spray-on approach offers an alternative route to conventional plastic mulch production and is particularly attractive for horticultural soil coverage.	[93]
2	Starch/PBAT-based biodegradable film	Films for strawberry cultivation were produced using blow extrusion, demonstrating the feasibility of thermoplastic processing for biodegradable agricultural mulch.	Starch blended with PBAT-type biodegradable polyester.	Demonstrated the potential of starch–polyester blends as processed mulch films for crop production, combining renewable starch with a flexible biodegradable polyester matrix.	[94]
3	PLA/PHB blend film	PLA was blended with PHB and processed under industrial extrusion-blown-film conditions to prepare agricultural mulch film.	PHB was used as a blending polymer, together with a reactive compatibilizing approach involving maleic-anhydride chemistry.	The work demonstrated that modification of inherently brittle PLA can improve its processability for industrial-scale blown mulch-film manufacture.	[95]
4	PLA/PHB biodegradable mulch film	A PLA/PHB blend was fabricated as a blown film for field mulching.	Here, 3 wt% carbon black was incorporated into the polymer blend to provide opacity and modify the environmental aging behavior of the mulch film.	The black biodegradable film was evaluated during a complete cultivation season in sweet pepper production, illustrating the use of carbon black for functional agricultural mulch formulations.	[96]
5	PBAT/OMMT nanocomposite film study	PBAT nanocomposite films were prepared through film blowing and biaxial orientation.	Organically modified montmorillonite (OMMT), at different loadings up to 13 wt%, was dispersed within PBAT.	Nanoclay incorporation was investigated primarily to overcome the relatively poor water-vapor barrier performance of neat PBAT, an important requirement for soil-moisture conservation in agricultural films.	[97]
6	PBAT-based reinforced nanocomposite	PBAT was compounded with two complementary nano-reinforcements to obtain a mechanically strengthened biodegradable composite.	Epoxide-modified nano silica and amine-treated cellulose nanofibers were incorporated to create interactive nano-reinforcement.	The combined nanofillers improved reinforcement and dispersion within the PBAT matrix, illustrating a potential approach for producing thinner but mechanically durable biodegradable agricultural films.	[98]
7	PBAT/PPC biodegradable blend film	PBAT and polypropylene carbonate (PPC) blends were prepared through reactive extrusion followed by blown-film processing.	EMA-GMA was incorporated as a reactive compatibilizer.	Compatibilization improved processing stability and influenced blend morphology, while also enhancing mechanical, gas-barrier, and UV-resistance characteristics relevant to exposed agricultural films.	[99]
8	PBAT/PLA biodegradable mulch film	Commercial PBAT/PLA biodegradable masterbatch was processed using a laboratory blown-film machine.	Commercial biodegradable PBAT/PLA masterbatch.	Demonstrated a comparatively direct blown-film route for manufacturing biodegradable mulch suitable for subsequent degradation and agricultural evaluation.	[100]
9	Plasticized PLA with biomass-derived fillers	Plasticized PLA-based films were formulated and converted into mulch-film specimens containing renewable particulate fillers.	Vegetable-derived fillers including spent coffee-type and plant-residue materials were incorporated at approximately 20 wt% in selected formulations.	Biomass fillers were explored as a means of reducing material cost and improving soil biodegradation while maintaining suitable mechanical, optical, and barrier performance.	[101]
10	PHA/PCL dual-layer film	A dual-layer mulch film was prepared by hot pressing. The first layer consisted of PHA, while the second contained PHA/PCL blends at ratios such as 90:10, 70:30, and 50:50. The layers were subsequently subjected to electron-beam irradiation.	PHA and PCL, electron-beam radiation was used to promote interlayer cross-linking/adhesion.	This is an important example of multilayer biodegradable-film engineering. The approach combines polymers with different characteristics and uses radiation cross-linking to improve adhesion between layers.	[102]
11	PBAT/PLA/chain-extender composite	A two-step extrusion process was employed. PBAT and PLA were first compounded in a twin-screw extruder, followed by pilot-scale extrusion of the optimized formulation.	PBAT/PLA at an optimized 80:20 ratio, with 1 phr chain extender.	Chain extension improved compatibility, rigidity, and strength. Importantly, the study demonstrated scale-up from laboratory twin-screw extrusion to pilot-scale production, making it particularly relevant to commercial agricultural mulch manufacturing.	[90]
12	Reactively compatibilized PLA/PBAT film	PLA and PBAT were subjected to reactive blending to improve the interface between the otherwise poorly compatible polymers.	Epoxidized soybean oil (ESO) was used as a reactive compatibilizer, and an epoxy-functionalized styrene-acrylic copolymer (ESA) as a chain extender. An optimized 75:25 PLA/PBAT formulation contained 5 phr ESO and 0.5 phr ESA.	The preparation strategy improved mechanical, thermal, and barrier characteristics and produced films suitable for extended field evaluation. It illustrates how bio-derived compatibilizers and chain extenders can be used to tailor agricultural film properties.	[103]
13	Alginate-based biodegradable mulch film	An alginate film was engineered using a dual-network strategy rather than relying on a single polysaccharide network.	Cross-linking/network-forming components were introduced to strengthen the alginate structure.	The dual-network approach was designed specifically to address the inadequate mechanical strength that frequently limits purely polysaccharide-based mulch films.	[104]
14	PBAT/PGA biodegradable blend	PBAT and polyglycolic acid were formulated as biodegradable blend films with stabilization against premature hydrolysis.	Antihydrolysis agents were investigated as formulation modifiers.	The strategy aimed to provide a more stable service life together with adequate mechanical performance, addressing the need for films that remain intact during cultivation but degrade after their useful period.	[105]
15	PBST/PGA biodegradable film	The polymers were subjected to reactive melt blending, using reactive compatibilization to improve processability; the optimized material was subsequently processed into mulch film for field testing.	Multifunctional epoxy oligomer and diisocyanate-type reactive agents were used for compatibilization.	The formulation targeted a combination of strength, barrier performance, and resistance to UV aging, demonstrating the importance of reactive processing for next-generation biodegradable mulch films.	[106]
16	PVA/starch biodegradable film	PVA–starch films were fabricated by electrospinning, creating continuous fibrous film structures without chemical modification.	Starch was incorporated into a PVA matrix; no chemical reinforcement was used.	The electrospun films exhibited moderate mechanical performance and survived for about 30 days in the preliminary study. This represents an alternative to conventional extrusion or solution-casting approaches.	[107]
17	Vegetable-stalk/sodium-alginate film	Untreated vegetable stalk material was directly combined with sodium alginate using a simple blending process, avoiding energy-intensive pretreatment.	Vegetable stalk (optimized around 30%), sodium alginate solution, and glycerol plasticizer.	The approach converts agricultural waste directly into biodegradable film. The optimized material exhibited mechanical, moisture-retention, and high UV-blocking performance and degraded rapidly after burial in moist soil.	[88]
18	PBAT/straw-lignocellulose nanosheet composite	Straw was converted into micro-/nanoscale lignocellulose sheets through a one-step solvent-free mechanochemical process, followed by incorporation of the nanosheets into PBAT.	Hydrophobic lignocellulose nanosheets retaining lignin.	The nanosheets improved dispersion in PBAT, reduced water-vapor transmission, and provided strong UV shielding. This demonstrates how agricultural residues can serve simultaneously as reinforcement, barrier modifiers, and natural UV-protective fillers.	[108]
19	PBAT/PPCP composite film	PBAT was compounded with different proportions of PPCP to prepare biodegradable composite mulching films.	PPCP with an effective formulation containing about 30 wt% PPCP.	The composite formulation substantially improved water-vapor barrier behavior and weathering durability and was subsequently evaluated in cotton cultivation.	[109]
20	PBAT/PPC-P/ADR film	Films were manufactured through one-step melt blending followed by blown-film processing.	PPC-P was used as a polymer modifier, and an ADR epoxy chain extender as an in situ reactive compatibilizer.	Reactive compatibilization improved melt-flow behavior and blown-film stability while enhancing strength and barrier characteristics, demonstrating a scalable route for high-performance biodegradable films.	[110]
21	Dead-leaf-derived lignocellulosic nanocellulose/PVA film	An all-water-based fabrication process was developed using nanocellulosic material derived from dead leaves and PVA, exploiting intermolecular hydrogen-bonding interactions to construct the film network.	Dead-leaf-derived lignocellulosic nanocellulose (LANC) and PVA.	The approach combines waste-biomass valorization with an aqueous manufacturing route. The resulting films showed measurable soil biodegradation and negligible adverse effects in the reported plant-germination/growth assessment.	[111]
22	PLA/ATBC/lignin agricultural film	PLA films were formulated by incorporating a biodegradable plasticizer and bio-derived UV absorber into the polymer matrix.	Acetyl tributyl citrate (ATBC) as plasticizer and kraft lignin as UV absorber; 12% ATBC was reported as a favorable plasticization level, while small lignin additions provided UV blocking.	This approach directly addresses two limitations of neat PLA for agricultural films: brittleness and vulnerability to solar UV. Lignin provides a renewable route to UV protection, while ATBC improves film flexibility.	[112]

**Table 4:** Comparative assessment of selected agro-industrial biomass resources within the proposed analytical framework for biodegradable packaging and plasticulture applications.

Material System	Main Material Role	Key Advantage	Main Limitation	Best-Suited Application	Industrial Maturity
Cassava/potato starch	Film-forming matrix	Low cost, established processing	Moisture sensitivity	Packaging, mulch films	Relatively high
Sweet potato	Matrix + pigment source	Film formation + bioactivity	Moisture sensitivity	Active/intelligent films	Moderate
Topinambur	Fructan hydrocolloid + bioactive source	Whole-plant valorization	Limited film/pigment validation	Active/intelligent packaging	Emerging
Yerba mate	Reinforcement + bioactive additive	Antioxidant, UV, multifunctionality	Extract variability, moisture effects	Active composites	Moderate
Rosehip	Bioactive/hydrophobic additive	Strong antioxidant potential	Matrix compatibility	Active packaging	Emerging–moderate
*Aloe vera*	Hydrocolloid + bioactive component	Edibility, antimicrobial activity	Weak moisture/mechanical resistance	Edible coatings	Moderate
Starch/PBAT	Biodegradable matrix blend	Renewable fraction + PBAT flexibility/processability	Moisture sensitivity; blend compatibility	Agricultural mulch films; flexible biodegradable packaging	Blown-film/field relevant
PLA/PHB	Biodegradable polyester blend	Improved renewable/biodegradable content	PLA brittleness; compatibilization needed	Agricultural mulch films; rigid/flexible biodegradable packaging	Industrial blown-film demonstrated
PBAT/OMMT	Nanocomposite matrix	Improved moisture barrier	Nanofiller dispersion/loading	Agricultural mulch films requiring improved moisture retention	Film blowing demonstrated
PBAT/PLA + chain extender	Compatibilized biodegradable polyester blend	Improved strength and processability	Added compatibilizer/process complexity	High-performance agricultural mulch films	Pilot-scale demonstrated
Alginate films	Polysaccharide film-forming matrix	Renewable, water-processable	Poor wet/mechanical strength	Spray-on mulch coatings; short-duration horticultural applications	Primarily laboratory/emerging
Electrospun PVA/starch	Fibrous biodegradable composite matrix	Tailored fibrous structure	Limited large-area scalability	Specialized biodegradable films; functional coatings or controlled-release systems	Laboratory stage

**Table 5:** Literature review for natural pigment-based active and intelligent food-packaging systems.

S. No.	Natural Pigment/Source	Packaging Matrix	Preparation/Formulation Concept	Packaging Function	Food Application/Indicator Target	Key Findings	Key Limitation	Commercialization Potential	References
1	Anthocyanins from red cabbage (*Brassica oleracea*)	Chitosan/PVA	Anthocyanin extract incorporated into chitosan–PVA film	Active + intelligent; time–temperature/color indicator	Time–temperature history of packaged foods	Red-cabbage anthocyanins imparted color-responsive behavior to chitosan/PVA films, demonstrating their suitability as natural time–temperature indicators.	Light/heat/oxygen stability and migration	Moderate–high among natural indicators	[80]
2	Anthocyanins from purple sweet potato (*Ipomoea batatas*)	Agar/potato starch	Anthocyanin extract immobilized in an agar–potato starch matrix	Intelligent; pH-responsive freshness indicator	Pork/meat spoilage	Films exhibited visible pH-dependent color changes over pH 2–10 and changed from red toward green as pork spoilage progressed.	Stability and matrix dependence	Moderate	[142]
3	Curcumin + anthocyanins	Starch/PVA/glycerol	Both natural pigments immobilized in a starch–PVA film-forming matrix	Intelligent; dual-pigment pH sensor	Fish freshness	The combined natural pigments produced visually detectable pH responses, enabling non-destructive, real-time monitoring of fish freshness.	Additional processing; potentially slower response	Emerging–moderate	[82]
4	Anthocyanins from *Echium amoenum*	Bacterial cellulose	Anthocyanins immobilized within a bacterial-cellulose film	Intelligent; colorimetric pH indicator	Shrimp freshness/spoilage	The bacterial-cellulose indicator showed clear pH-induced color changes and successfully responded to freshness deterioration in shrimp.	Additional processing; potentially slower response	Emerging–moderate	[83]
5	Curcumin	Pectin + sulfur nanoparticles	Curcumin and sulfur nanoparticles dispersed in pectin by solution casting	Active + intelligent; antioxidant, antibacterial and pH/NH_3_-responsive	Shrimp spoilage	Curcumin provided pH/ammonia sensitivity, while sulfur nanoparticles contributed functional activity; the film showed antibacterial and antioxidant properties and a visible response to shrimp spoilage.	Restricted color-response range and photostability	Moderate	[50]
6	Anthocyanins	Gellan gum	Anthocyanins incorporated into gellan-gum films with controlled pigment release	Active + intelligent	Food-freshness monitoring	Gellan-gum-based films were designed to regulate anthocyanin release while simultaneously providing freshness-monitoring capability.	Additional processing; potentially slower response	Emerging–moderate	[145]
7	Shikonin from gromwell root	Gelatin/carrageenan + propolis	Shikonin and propolis incorporated into a gelatin–carrageenan matrix	Active + intelligent; pH-responsive and bioactive	Milk freshness	Shikonin supplied broad pH-responsive color change, while propolis contributed active functionality, producing a multifunctional smart-packaging film.	--	--	[146]
8	Barberry anthocyanins	Methylcellulose/chitosan nanofibers	Anthocyanin extract incorporated into a methylcellulose–chitosan nanofiber composite	Active + intelligent; antioxidant and pH-responsive	Lamb-meat freshness	The composite films exhibited colorimetric sensitivity to pH changes and were demonstrated for real-time monitoring of meat freshness.	Additional processing; potentially slower response	Emerging–moderate	[147]
9	Anthocyanins	Starch/PVA	Free versus nano-encapsulated anthocyanins incorporated into starch/PVA films	Active + intelligent	Bass-fillet freshness	Free anthocyanins provided stronger light-blocking and antioxidant effects, whereas encapsulation improved pigment stability and moisture-barrier performance; both approaches enabled freshness indication.	Additional processing; potentially slower response	Emerging–moderate	[148]
10	Anthocyanin-rich purple potato extract	TEMPO-oxidized bacterial cellulose + thymol	Anthocyanin-rich extract used as the sensing component and thymol as the active antimicrobial component	Active + intelligent; antimicrobial + freshness indicator	Shrimp shelf-life and freshness	The combination enabled simultaneous spoilage indication and active preservation, demonstrating the potential of integrating a natural pigment with an antimicrobial agent.	Stability and matrix dependence	Moderate	[149]
11	Blueberry anthocyanins	Corn starch/PVA + ovalbumin–CMC nanocomplexes	Anthocyanins first encapsulated in ovalbumin–carboxymethyl cellulose nanocomplexes and then incorporated into starch/PVA film	Intelligent; stabilized colorimetric indicator	Mushroom freshness	Nanocomplex encapsulation improved anthocyanin stability and enabled colorimetric monitoring of mushroom freshness.	Additional processing; potentially slower response	Emerging–moderate	[143]
12	Curcumin	Sodium cellulose sulfate/chitosan	Curcumin incorporated into an oppositely charged polysaccharide composite matrix	Active + intelligent; pH-responsive and preservation function	Pork freshness	The films responded visibly over pH 3–10, monitored pork freshness, and extended pork shelf life by approximately four days in the reported experiment.	Restricted color-response range and photostability	Moderate	[150]
13	Curcumin	Gelatin/chitosan nanofibers	Curcumin-loaded gelatin/chitosan nanofibers prepared by electrospinning	Active + intelligent; antioxidant, antibacterial and NH_3_-responsive	Protein-rich foods, meat and seafood	Curcumin improved antioxidant and antibacterial functionality; films containing 0.2% curcumin showed an ammonia-responsive color change within about 3 min.	Restricted color-response range and photostability	Moderate	[39]

## Data Availability

The raw data supporting the conclusions of this article will be made available by the authors on request.
